# ChemR23 activation attenuates cognitive impairment in chronic cerebral hypoperfusion by inhibiting NLRP3 inflammasome-induced neuronal pyroptosis

**DOI:** 10.1038/s41419-023-06237-6

**Published:** 2023-11-06

**Authors:** Yaxuan Zhang, Jiawei Zhang, Yao Zhao, Yueqi Zhang, Lan Liu, Xiaofeng Xu, Xiuzhe Wang, Jianliang Fu

**Affiliations:** 1 Department of Neurology, Shanghai Sixth People’s Hospital Affiliated to Shanghai Jiao Tong University School of Medicine, 600 Yishan Road, Shanghai, 200233 China; 2 Department of Neurology, Zhongshan Hospital, Fudan University, Shanghai, China

**Keywords:** Cell death in the nervous system, Cognitive neuroscience

## Abstract

Neuroinflammation plays critical roles in vascular dementia (VaD), the second leading cause of dementia, which can be induced by chronic cerebral hypoperfusion (CCH). NLRP3 inflammasome-induced pyroptosis, the inflammatory programmed cell death, has been reported to contribute to the development of VaD. ChemR23 is a G protein-coupled receptor that has emerging roles in regulating inflammation. However, the role of ChemR23 signalling in NLRP3 inflammasome-induced pyroptosis in CCH remains elusive. In this study, a CCH rat model was established by permanent bilateral common carotid artery occlusion (BCCAO) surgery. Eight weeks after the surgery, the rats were intraperitoneally injected with the ChemR23 agonist Resolvin E1 (RvE1) or chemerin-9 (C-9). Additionally, primary rat hippocampal neurons and SH-SY5Y cells were adopted to mimic CCH injury in vitro. Our results showed that the levels of ChemR23 expression were decreased from the 8^th^ week after BCCAO, accompanied by significant cognitive impairment. Further analysis revealed that CCH induced neuronal damage, synaptic injury and NLRP3-related pyroptosis activation in hippocampal neurons. However, pharmacologic activation of ChemR23 with RvE1 or C-9 counteracted these changes. In vitro experiments also showed that ChemR23 activation prevented primary neuron pyroptosis induced by chronic hypoxia. In addition, manipulating ChemR23 expression markedly regulated NLRP3 inflammasome-induced neuronal pyroptosis through PI3K/AKT/Nrf2 signalling in SH-SY5Y cells under hypoglycaemic and hypoxic conditions. Collectively, our data demonstrated that ChemR23 activation inhibits NLRP3 inflammasome-induced neuronal pyroptosis and improves cognitive function via the PI3K/AKT/Nrf2 signalling pathway in CCH models. ChemR23 may serve as a potential novel therapeutic target to treat CCH-induced cognitive impairment.

## Introduction

The second most common cause of dementia, vascular dementia (VaD), which is characterized by progressive cognitive impairment and memory loss has become a major public health challenge [1]. Among all the underlying mechanisms, CCH plays a critical role by inducing neuroinflammation, oxidative stress and neuronal pyroptosis, eventually leading to synaptic plasticity impairment and cognitive impairment [2–5].

Pyroptosis is a pro-inflammatory form of cell death initiated by inflammasomes involved in various neurological disorders, including Alzheimer’s disease (AD) and stroke [6, 7]. The assembly of the inflammasome begins with the oligomerization of cytosolic particular pattern recognition receptors, including NOD-, LRR-, and pyrin domain-containing protein 3 (NLRP3), followed by the recruitment of apoptosis-associated speck-like protein containing a CARD (ASC) and autoactivation of caspase-1. Activated caspase-1 further processes interleukin (IL) ‐1β, IL‐18 and gasdermin D (GSDMD) [8]. GSDMD-N (the active form of GSDMD) will then induce the rupture of the cell membrane and leakage of inflammatory cellular contents, ultimately leading to pyroptosis and inflammation [8]. NLRP3 inflammasome-mediated pyroptosis has been reported to contribute to exacerbated neuroinflammation in BCCAO rats, a rodent model of VaD [5, 9]. Targeting NLRP3 inflammasome-mediated pyroptosis may provide new insights into the development of novel therapeutic methods for CCH-induced VaD.

ChemR23, also known as chemokine-like receptor 1, is a G-protein coupled receptor that is ubiquitously expressed in neurons and glial cells [10]. Previous studies demonstrated that ChemR23 was involved in the pathogenesis of chronic inflammatory diseases, such as atherosclerosis, inflammatory bowel disease and AD [11–13]. The levels of ChemR23 were significantly increased in the brains of AD patients and correlated with Braak pathological staging, suggesting that ChemR23 might be involved in the pathogenesis of dementia [10, 13]. However, the role of ChemR23 in CCH-related cognitive impairment remains elusive.

RvE1 and chemerin are both endogenous agonists of ChemR23 and have been implicated in the regulation of the inflammatory process. RvE1, a derivative of eicosapentaenoic acid, belongs to the superfamily of specialized pro-resolving mediators, which are the key players in promoting the resolution of inflammation [14–16]. Additionally, chemerin, an adipokine that undergoes multiple proteolytic cleavages to generate different variants, has also been proven to regulate inflammatory responses [16, 17]. C-9 is the most bioactive fragment of chemerin [17] and has been reported to exert an anti-inflammatory effect [18] and improve the memory deficits induced by amyloid β [19].

In the present study, we investigated the expression pattern of ChemR23 in the brain of CCH rat model and explored the treatment effects of the ChemR23 agonists, RvE1 and C-9. Furthermore, by using cellular models, we explored the underlying mechanisms of ChemR23 signalling in CCH.

## Results

### Expression pattern of ChemR23 in the hippocampus after CCH

**Fig. 1:**
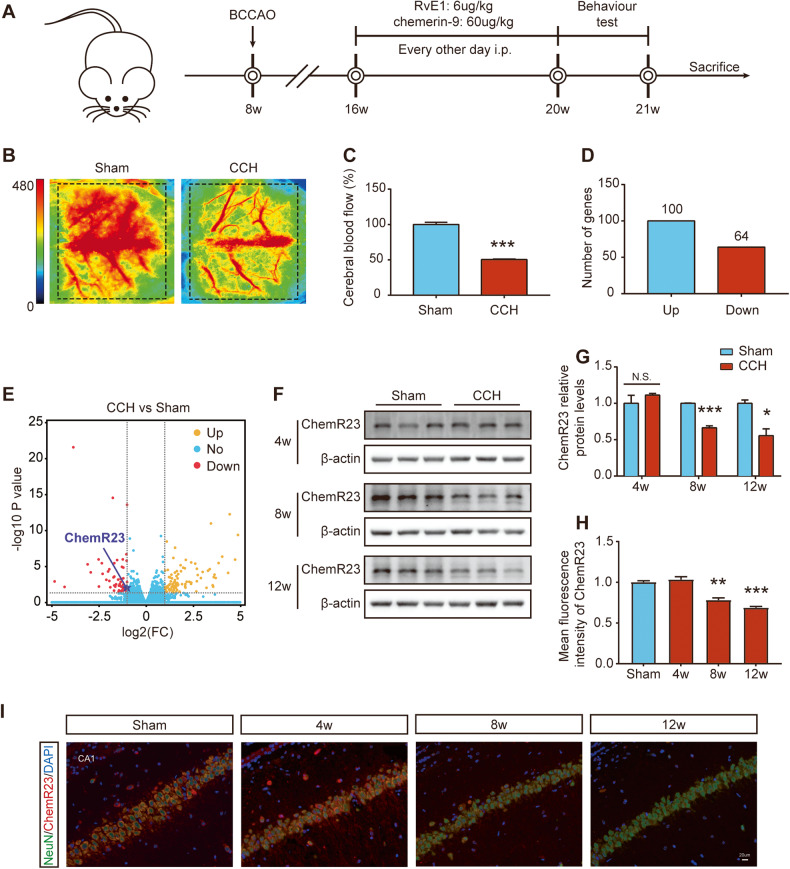
Expression pattern of ChemR23 in the rat hippocampus after CCH. **A** Schematic diagram of the experimental procedure. **B**, **C** Representative images and quantification of CBF before and after BCCAO surgery. *n* = 3 per group. **D**, **E** Total numbers of genes that were upregulated or downregulated and the volcano plot of DEGs between the CCH and Sham groups. *n* = 4 per group. **F**, **G** Representative immunoblotting bands and semiquantitative analysis of ChemR23 in the hippocampus at different time points after BCCAO. *n* = 3 per group. **H** Mean fluorescence intensity of ChemR23. *n* = 3 per group. **I** Representative merged immunofluorescence images of NeuN, ChemR23 and DAPI in the hippocampal CA1 region. Scale bar: 20 μm. *n* = 3 per group. Data are presented as the mean ± SEM. *P < 0.05, **P < 0.01, ***P < 0.001 vs. sham.

We successfully established a CCH rat model by performing BCCAO surgery, in which the cerebral blood flow (CBF) decreased significantly to approximately 50% of the baseline (Fig. 1B, C). Our previous studies reported that the BCCAO model induced cognitive impairment from the 8th week after surgery, and neuronal damage in the hippocampal CA1 region was also observed at the same time [20]. Therefore, we used rats at the 8th week after surgery to perform RNA-seq analysis in the sham and CCH groups. The results showed that a total of 100 and 64 genes were upregulated or downregulated, respectively (Fig. 1D), and ChemR23 was among the significantly decreased genes in CCH rats (Fig. 1E). We also performed western blot to evaluate the expression of ChemR23 in the hippocampal regions at 4, 8 and 12 weeks. As shown in Fig. 1F and G, the protein levels of ChemR23 were significantly decreased at 8 and 12 weeks, while no significant fluctuations were observed at 4 weeks. To further explore the expression levels of ChemR23 in different nerve cells, double immunofluorescence staining of ChemR23/NeuN, ChemR23/Iba-1 and ChemR23/GFAP was performed on brain sections. The results showed that ChemR23 was mainly expressed in neurons, but was expressed at relatively low levels in microglia and astrocytes (Fig. S1A–C). Furthermore, the fluorescence intensity of ChemR23 in hippocampal neurons in the CCH group was weaker than that in the sham group at 8 and 12 weeks after the operation (Fig. 1H, I, S2A). The results above suggest a possible association between cognitive function and ChemR23 signalling in CCH rats.

### ChemR23 activation by RvE1 or C-9 attenuated the cognitive impairment induced by CCH

**Fig. 2:**
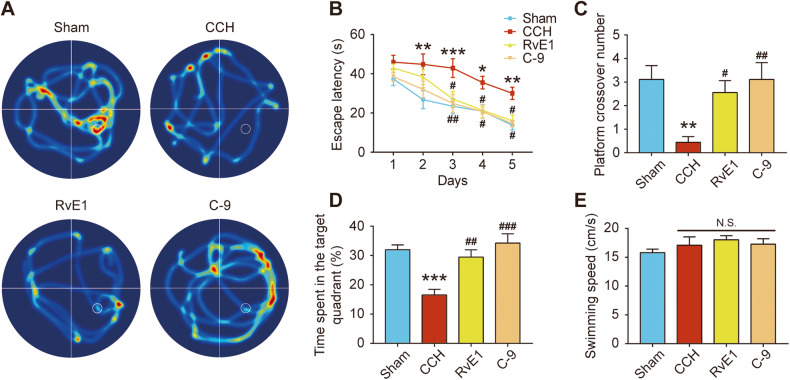
ChemR23 activation by RvE1 or C-9 improved cognitive impairment in CCH rats. **A** Representative exploring traces of rats in different groups during the probe trial. **B** The escape latency of rats to reach the platform during the acquisition test. *n* = 9 per group. **C** The numbers of platform crossings in the probe trial. *n* = 9 per group. **D** Percentage of time spent in the 3rd quadrant of rats in the probe trial. *n* = 9 per group. **E** Average swimming speeds of rats in different groups during the probe trial. *n* = 9 per group. Data are presented as the mean ± SEM. *P < 0.05, **P < 0.01, ***P < 0.001 vs. sham; #P < 0.05, ##P < 0.01, ###P < 0.001 vs. CCH; N.S. means not significant.

To determine how ChemR23 activation affected the cognitive performance of CCH rats, we conducted the MWM test after the administration of RvE1 or C-9 for 4 weeks. As shown in Fig. 2B, the escape latency in the CCH group was significantly prolonged compared to that in the sham group from Day 2, while treatment with RvE1 or C-9 significantly reduced the latency. After the spatial acquisition training, we removed the platform and performed the probe test. Treatment with RvE1 or C-9 increased the time spent in the target quadrant and elevated the frequency of platform crossings (Fig. 2A, C, D). Additionally, there was no significant difference in swimming speed among all groups (Fig. 2E), indicating no significant visual or motior abnormality among the rats. Altogether, these results demonstrated that the ChemR23 agonists RvE1 and C-9 were protective against CCH-induced cognitive dysfunction.

### ChemR23 activation with RvE1 and C-9 ameliorates neuronal damage and synaptic injury in CCH rats

**Fig. 3:**
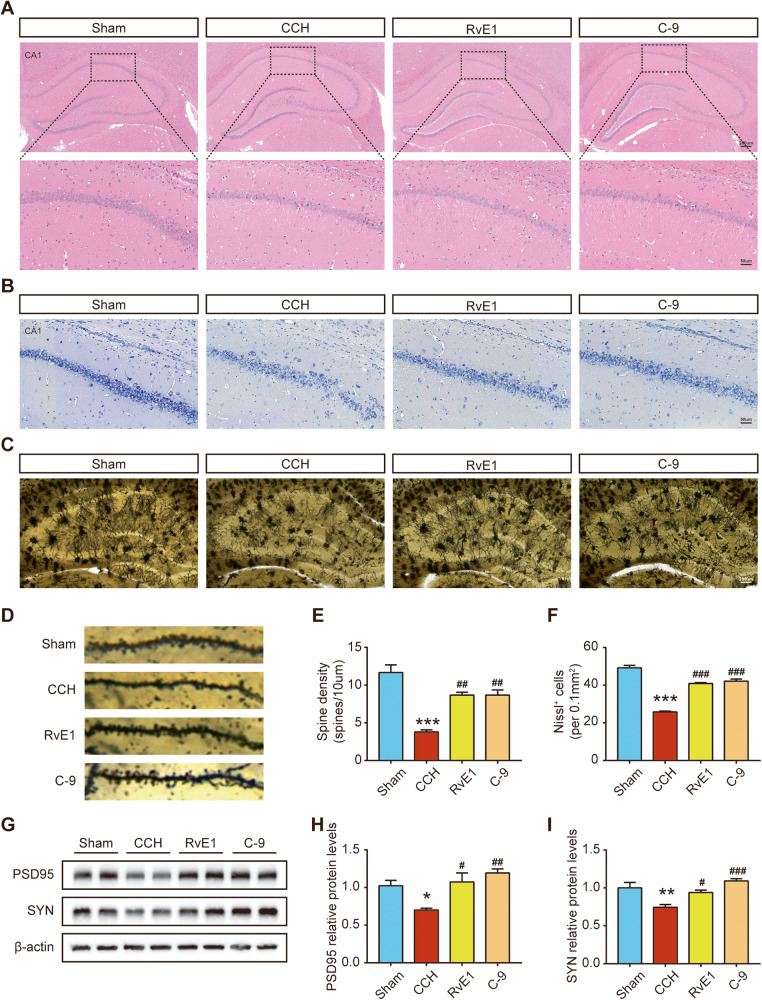
Effects of ChemR23 activation with RvE1 or C-9 on neuronal damage and synaptic dysfunction in CCH rats. **A** Representative H&E staining of the rat hippocampus. Scale bar: 200 μm (upper panel) or 50 μm (lower panel). *n* = 3 per group. **B** Representative Nissl staining in the rat hippocampus. Scale bar: 50 µm. *n* = 3 per group. **C** Golgi staining in the rat hippocampus. Scale bar: 200 μm. *n* = 3 per group. **D**, **E** Enlarged images of neuronal dendrites by Golgi staining and quantification of spine density. *n* = 3 per group. **F** Quantification of Nissl-positive cells in the rat hippocampus. *n* = 3 per group. **G**–**I** Representative immunoblotting bands and semiquantitative analysis of SYN and PSD95 in the rat hippocampus. *n* = 4 per group. Data are presented as the mean ± SEM. *P < 0.05, **P < 0.01, ***P < 0.001 vs. sham; #P < 0.05, ##P < 0.01, ###P < 0.001 vs. CCH.

Cognitive impairment was associated with neuronal histopathological abnormalities and impairment of synaptic plasticity in CCH [21, 22]. Therefore, we performed H&E and Nissl staining on hippocampal sections. As shown by the micrographs of H&E staining (Fig. 3A), the neurons in sham rats appeared normal and regularly arranged with clear nuclei, while the neurons in CCH rats exhibited pathological changes, including karyopyknosis and deeper staining intensity. The above neuronal pathological changes were significantly improved by RvE1 or C-9 in the treatment groups. In addition, the results of Nissl staining showed that, compared with the sham group, the number of Nissl-positive neurons in the CCH group was reduced, and the arrangement was loosened, suggesting neuronal damage and degeneration. Additionally, treatment with RvE1 or C-9 rescued these changes (Fig. 3B, F). Furthermore, we analysed the expression of synapse-related proteins and dendritic spine density to assess synaptic plasticity in the hippocampus. As shown in Fig. 3G–I, compared to those in the sham group, the relative expressions levels of PSD95 and SYN were significantly reduced in CCH rats, while administration of RvE1 or C-9 reversed this decrease. Consistent with the western blot results, immunofluorescence staining of PSD-95 and SYN revealed increased expression of PSD-95 and SYN in the hippocampal CA1 region after RvE1 or C-9 treatment (Fig. S3A–D). In addition, as shown by Golgi staining, the dendritic spine density of neurons in the RvE1 and C-9 treatment groups was increased compared to that in the vehicle-treated CCH group (Fig. 3C–E). Cumulatively, these results suggested that treatment with RvE1 or C-9 prevented neuronal morphological abnormalities and impairment of synaptic plasticity in CCH rats.

### ChemR23 activation with RvE1 or C-9 inhibited neuronal pyroptosis after CCH

**Fig. 4:**
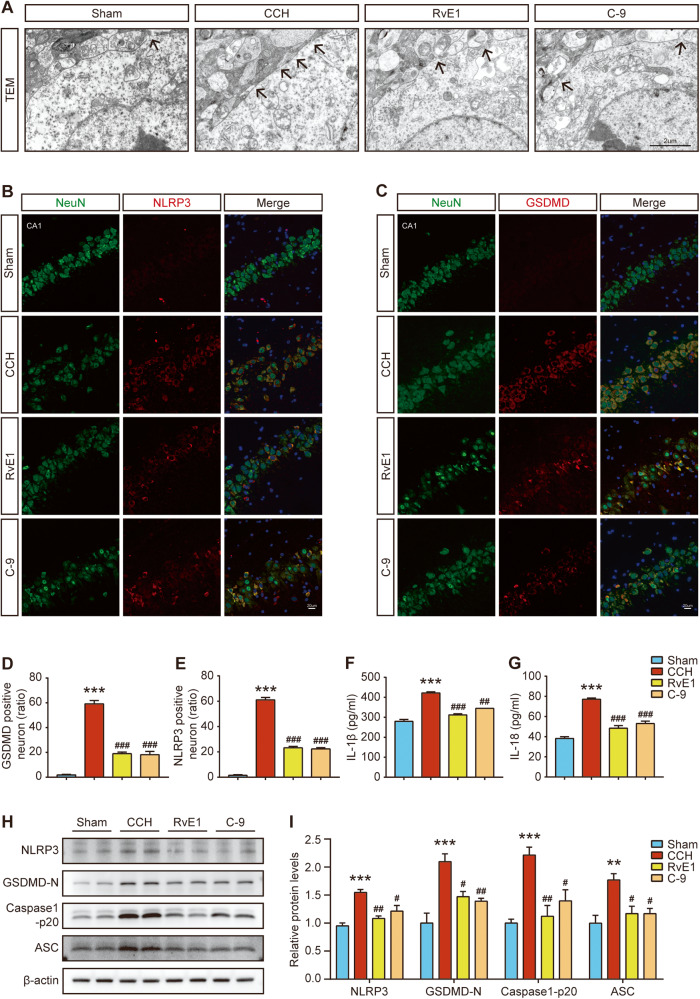
Effects of ChemR23 activation with RvE1 or C-9 on neuronal pyroptosis in CCH rats. **A** Representative transmission electron micrographs of hippocampal neurons in different treatment groups. Black arrowhead: membrane pores. Scale bar: 2 μm. *n* = 3 per group. **B**, **C** Representative double immunofluorescence staining of GSDMD/NeuN and NLRP3/NeuN in the rat hippocampus. Scale bar: 20 μm. *n* = 3 per group. **D**, **E** Quantitative statistics of GSDMD/NeuN or NLRP3/NeuN double-positive cells in rat hippocampus. *n* = 3 per group. **F**, **G** ELISA results of IL-1 and IL-18 analysis in the rat hippocampus. *n* = 4 per group. **H**, **I** Representative immunoblotting bands and the expression levels of NLRP3, GSDMD-N, Caspase-1 p20 and ASC in the rat hippocampus. *n* = 4 per group. Data are presented as the mean ± SEM. **P < 0.01, ***P < 0.001 vs. sham; #P < 0.05, ##P < 0.01, ###P < 0.001 vs. CCH.

Next, we sought to explore whether neuronal damage and synaptic injury were associated with pyroptosis in CCH rats and whether ChemR23 activation plays a role therein. Pyroptosis is a lytic form of cell death that relies on GSDMD-induced membrane pore formation. Therefore, TEM was used to observe the changes in the neuronal membrane after CCH. As shown in Fig. 4A, the number of membrane pores on hippocampal neurons was markedly increased in CCH rats, while RvE1 or C-9 administration mitigated this abnormality. In addition, double immunofluorescence staining of NLRP3/NeuN and GSDMD/NeuN was performed on brain sections. The results suggested that, compared to the sham group, the numbers of NLRP3- or GSDMD-positive neurons were markedly increased in CCH rats; however, RvE1 or C-9 treatment prevented the increase in these neurons (Fig. 4B–E). Subsequently, western blot and ELISA were used to further evaluate the expression of pyroptosis-related proteins in the hippocampus. As depicted in Fig. 4H and I, the expression levels of NLRP3, GSDMD‐N, caspase-1 p20 (the active form of caspase‐1) and ASC were dramatically increased in CCH rats, and these increases were reversed by treatment with RvE1 or C-9. Consistently, the ELISA results also revealed that the levels of IL-1β and IL-18 in CCH rats were significantly reduced after RvE1 or C-9 treatment (Fig. 4F, G). Taken together, these data support the notion that CCH triggers pyroptotic cell death in neurons and further confirm the important role of ChemR23 activation in ameliorating CCH-induced neuronal pyroptosis. It was previously established that abnormal autophagy and apoptosis are crucial for CCH-related brain damage [23]. Therefore, to further explore the effects of ChemR23 activation, we analysed the expression of apoptosis- and autophagy-related proteins. As presented in Fig. S4A–C, CCH significantly decreased the expression of Bcl-2 and increased the expression of Bax, suggesting the occurrence of apoptosis. RvE1 or C-9 treatment partially reversed the changes in Bcl-2 and Bax expression in CCH rats, but statistical analysis showed no significant difference. In addition, the levels of the autophagy-related proteins LC3B and p62 were also detected by western blot. The results showed that RvE1 and C-9 treatment significantly reduced CCH-induced increases in LC3B-II and P62 expression, indicating that ChemR23 activation attenuated the dysfunction of autophagy in CCH [Fig. S4D–F].

### ChemR23 activation with RvE1 and C-9 regulated the PI3K/AKT/Nrf2 signalling pathway in CCH rats

**Fig. 5:**
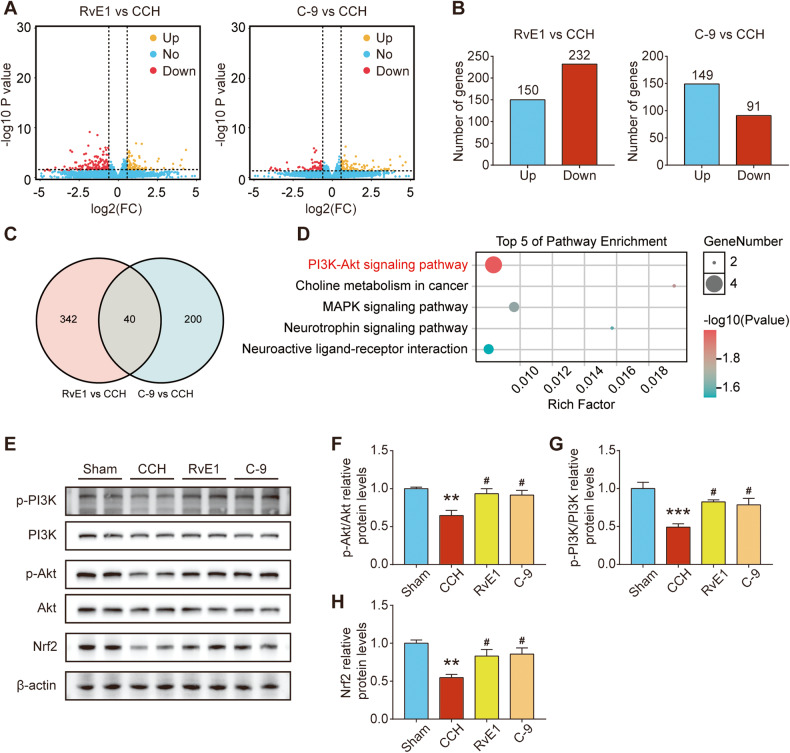
ChemR23 activation with RvE1 or C-9 regulated PI3K/AKT/Nrf2 signalling pathway in CCH rats. **A** Volcano plots of DEGs of the RvE1 group vs. the CCH group and the C-9 group vs. the CCH group. *n* = 3 per group. **B** Total numbers of upregulated or downregulated genes in the RvE1 group vs. the CCH group and the C-9 group vs. the CCH group. *n* = 3 per group. **C** A Venn diagram displaying the intersection of DEGs for RvE1 vs. CCH and C-9 vs. CCH. *n* = 3 per group. **D** The intersection of DEGs for RvE1 vs. CCH and C-9 vs. CCH was analysed by KEGG enrichment and the top 5 enriched pathways are shown. *n* = 3 per group. **E**–**H** Representative immunoblotting bands and the levels of p-PI3K/PI3K, p-AKT/AKT and Nrf2 in the rat hippocampus. *n* = 4 per group. Data are presented as the mean ± SEM. **P < 0.01, ***P < 0.001 vs. sham; #P < 0.05 vs. CCH.

To investigate the potential mechanisms by which ChemR23 activation regulates neuronal pyroptosis, RNA-seq analysis was performed on hippocampal tissues from the CCH- and RvE1/C-9-treated groups. The differentially expressed genes (DEGs) of RvE1- or C-9- treated groups were screened out as those with a fold change ≥ 1.5 and p value ≤ 0.05 compared with the CCH group (Fig. 5A, B). KEGG pathway enrichment analysis of the overlapping DEGs among RvE1 vs. CCH and C-9 vs. CCH indicated that the pathway most affected by ChemR23 activation is the PI3K-AKT signalling pathway (Fig. 5C, D). We further confirmed this finding by immunoblotting. As shown in Fig. 5E–G, the expression levels of p-PI3K/PI3K and p-AKT/AKT were markedly increased in RvE1- or C-9-treated CCH rats compared to vehicle-treated CCH rats. Moreover, Nrf2 has attracted considerable attention for its inhibition of NLRP3 inflammasome activation, and PI3K/Akt signalling is a vital upstream regulator of Nrf2 [24–26]. Thus, we subsequently evaluated the levels of Nrf2 by western blot analysis. The results suggested that treatment with RvE1 or C-9 also significantly reversed the decrease in Nrf2 expression in CCH rats (Fig. 5E, H). Based on these findings, we concluded that the PI3K/AKT/Nrf2 axis appeared to be a key mechanism in ChemR23 activation by RvE1 and C-9 in CCH rats.

### ChemR23 activation inhibited pyroptosis induced by hypoxic stimulation in primary hippocampal neurons

**Fig. 6:**
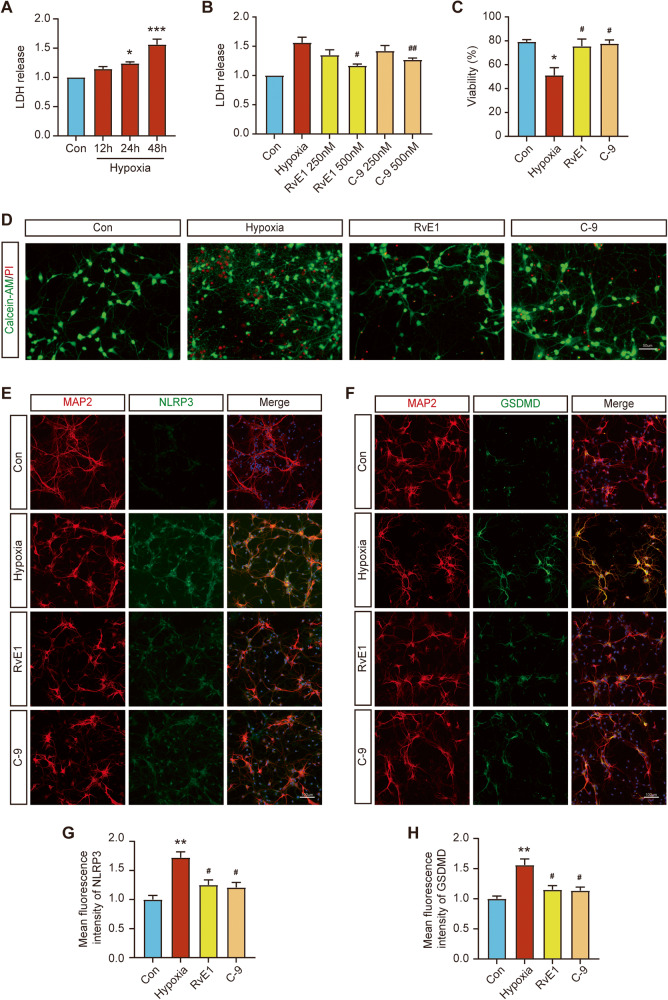
ChemR23 activation ameliorated pyroptosis after chronic hypoxic stimulation in primary rat hippocampal neurons. **A** Changes in LDH release in the cell culture supernatant with time under hypoxic conditions. *n* = 4 per group. **B** Effects of different concentrations of RvE1 and C-9 on the release of LDH in primary neurons under hypoxic stimulation. *n* = 4 per group. **C** Cell viability of each group under different treatment conditions. *n* = 3 per group. **D** Fluorescence images of primary neurons stained with calcein AM (live cells, green fluorescence) and PI (dead cells, red fluorescence) after different treatments. Scale bar: 50 μm. *n* = 3 per group. **E** Double immunostaining of NLRP3 with MAP2 in primary neurons of different groups. Scale bar: 100 μm. *n* = 3 for each group. **F** Double immunostaining of GSDMD with MAP2 in primary neurons of different groups. Scale bar: 100 μm. *n* = 3 for each group. **G** The statistics of the mean fluorescence intensity of NLRP3 in each group. *n* = 3 for each group. **H** The statistics of the mean fluorescence intensity of GSDMD in each group. *n* = 3 for each group. Data are presented as the mean ± SEM. *P < 0.05, **P < 0.01, ***P < 0.001 vs. Con; #P < 0.05, ##P < 0.01 vs. Hypoxia.

To confirm the protective effects of ChemR23 activation in primary hippocampal neurons, an experimental model of sustained hypoxic stimulation with 3% oxygen was established to mimic CCH in vitro. LDH assay kits and live-dead staining kits were used to detect the membrane integrity and cell viability of the cultured hippocampal neurons, respectively. As shown in Fig. 6A, LDH release increased with prolonged hypoxic stimulation. LDH release was significantly enhanced in the hypoxia group compared with the Con group at 48 h. Additionally, 48 h of hypoxic stimulation markedly reduced the viability of neurons (Fig. 6C, D). Therefore, the time point of 48 h of hypoxic stimulation was chosen to mimic CCH in subsequent experiments. Moreover, our results showed that LDH release was reduced by RvE1 or C-9 treatment, and the optimal concentration was 500 nM (Fig. 6B). Consistently, the results of live-dead staining assays showed that ChemR23 activation ameliorated the hypoxia-induced decline in cell viability (Fig. 6C, D). Next, we further analysed the expression levels of the pyroptosis-associated proteins NLRP3 and GSDMD by immunofluorescence staining. As shown in Fig. 6E–H, hypoxic stimulation increased the immunofluorescence intensity of NLRP3 and GSDMD in primary hippocampal neurons, while ChemR23 activation reversed these effects. These data suggested that ChemR23 activation exerted a protective effect by ameliorating neuronal pyroptosis induced by chronic hypoxia in vitro.

### ChemR23 activation inhibited pyroptosis induced by hypoglycaemic and hypoxic stimulation in SH-SY5Y cells

**Fig. 7:**
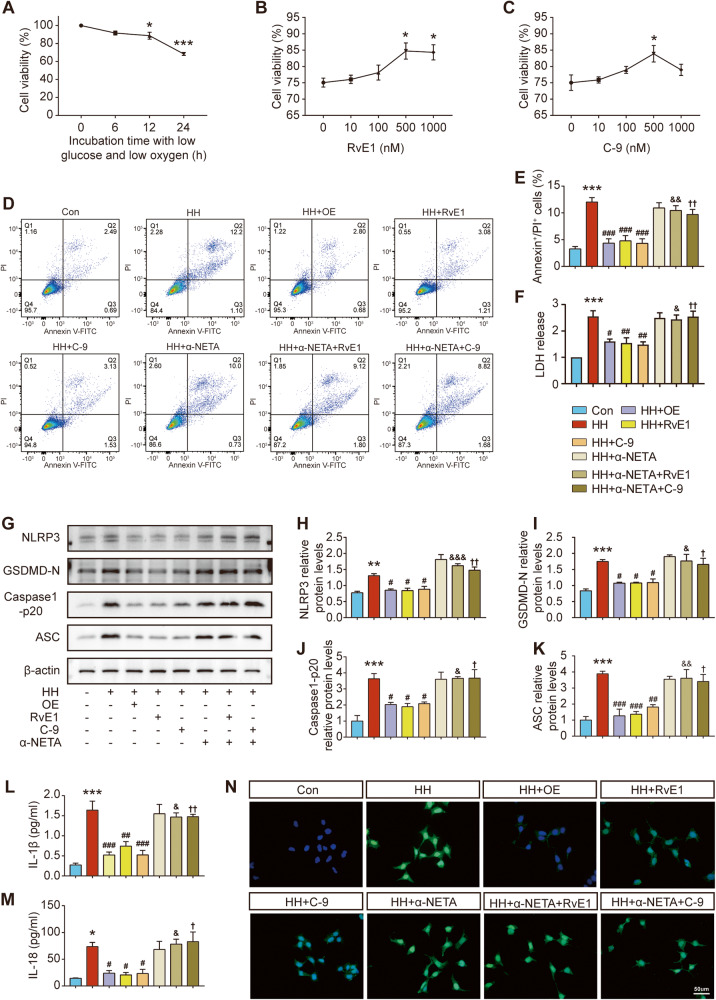
Effects of ChemR23 activation on pyroptosis in SH-SY5Y cells after hypoglycaemic and hypoxic stimulation. **A** Changes in cell viability with time under hypoglycaemic and hypoxic conditions. *n* = 4 per group. **B**, **C** Effects of different concentrations of RvE1 and C-9 on cell viability. *n* = 4 per group. **D**, **E** The proportion of Annexin V/PI double positive cells analysed by flow cytometry. *n* = 3 per group. **F** LDH levels in the cell culture supernatant. *n* = 3 per group. **G**–**K** Western blot analysis of NLRP3, GSDMD-N, Caspase-1 p20 and ASC. *n* = 3 per group. **L**, **M** Concentrations of IL-1β and IL-18 in the cell culture supernatant were measured by ELISA. *n* = 4 per group. (N) Representative immunofluorescence staining of NLRP3 (green) with the nuclei counterstained by DAPI. Scale bar: 50 μm. *n* = 3 per group. Data are presented as the mean ± SEM. **P < 0.01, ***P < 0.001 vs. Con; #P < 0.05, ##P < 0.01, ###P < 0.001 vs. HH; &P < 0.05, &&P < 0.01, &&&P < 0.001 vs. HH+RvE1; †P < 0.05, ††P < 0.01, †††P < 0.001 vs. HH + C-9.

SH-SY5Y cells cultured with 3% oxygen +1 g/l glucose medium were used to mimic chronic cerebral hypoperfusion in vitro. First, the optimal hypoxia and hypoglycaemia culture times of SH-SY5Y cells and the optimal dose concentrations of RvE1 and C-9 were determine. A CCK-8 assay was used to analyse cell viability. As shown in Fig. 7A, the cell viability markedly decreased with time. When the modelling time continued to 24 h, the cell viability dropped to 68%. Therefore, the time point of 24 h was selected as the optimal modelling time. Moreover, RvE1 or C-9 at a concentration of 500 nM proved to have the most neuroprotective effects (Fig. 7B, C) on cell viability, and a 500 nM concentration was therefore chosen for further study. Subsequently, SH-SY5Y cells were overexpressed with ChemR23 and exposed to ChemR23 antagonist α-NETA to study the effects of activation or inhibition of ChemR23 signalling. The results showed that RvE1 or C-9 treatment and ChemR23 overexpression reduced the increase in LDH release and the number of Annexin V/PI double-positive cells following hypoglycaemia and hypoxic stimulation, and the protective effects of RvE1 or C-9 were abolished by the ChemR23 antagonist α-NETA (Fig. 7D–F). In addition, consistent with the in vivo results, the levels of NLRP3, GSDMD-N, ASC, caspase-1 p20, IL-1β and IL-18 in the cell culture supernatant were strikingly decreased by RvE1 and C-9 treatments, while α-NETA treatment abrogated these curative effects (Fig. 7G–M). Additionally, the expression of NLRP3 was further confirmed by immunofluorescence staining. As shown in Fig. 7N, the expression pattern of NLRP3 shown by immunofluorescence staining was similar to the western blot results. All these results demonstrate the protective effects of ChemR23 activation in regulating neuronal pyroptosis following hypoglycaemic and hypoxic stimulation under in vitro conditions.

### ChemR23 rescued SH-SY5Y pyroptosis induced by hypoglycaemic and hypoxic stimulation via the PI3K/AKT/Nrf2 signalling pathway

**Fig. 8:**
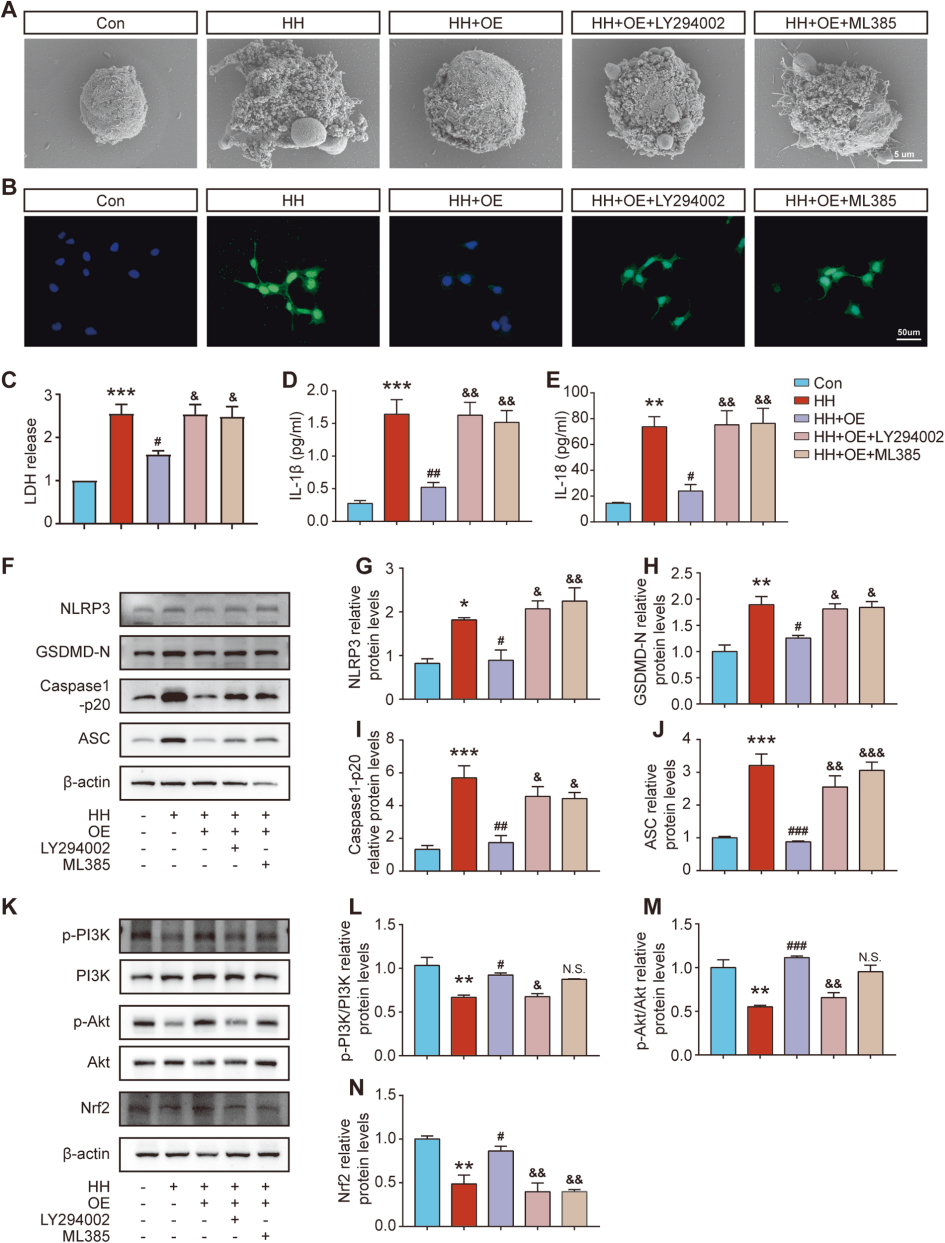
ChemR23 activation inhibited neuronal pyroptosis via the PI3K/AKT/Nrf2 signalling pathway in SH-SY5Y cells. **A** Scanning electron microscopy analysis showing the cell morphology in different treatment groups. *n* = 3 per group. **B** Representative immunofluorescence staining of NLRP3 (green) with the nuclei counterstained by DAPI. Scale bar: 50 μm. *n* = 3 per group. **C** LDH levels in the cell culture supernatant. *n* = 3 per group. **D**, **E** ELISA of IL-1β and IL-18 levels. *n* = 3 per group. **F**–**J** The levels of NLRP3, GSDMD-N, Caspase-1 p20 and ASC were evaluated by western blot. *n* = 3 per group. **K**–**N** The expression levels of p-PI3K/PI3K, p-AKT/AKT and Nrf2 were evaluated by western blot. *n* = 3 per group. Data are presented as the mean ± SEM. *P < 0.05, **P < 0.01, ***P < 0.001 vs. Con; #P < 0.05, ##P < 0.01, ###P < 0.001 vs. HH; &P < 0.05, &&P < 0.01, &&&P < 0.001 vs. HH + OE; N.S. means not significant vs. HH + OE.

To explore whether ChemR23 regulated neuronal pyroptosis through the PI3K/AKT/Nrf2 signalling pathway in vitro, SH-SY5Y cells overexpressing ChemR23 were exposed to the PI3K inhibitor LY294002 and the Nrf2 inhibitor ML385 after hypoglycaemic and hypoxic stimulation. SEM was used to observe the morphological changes in SH-SY5Y cells. The results showed that the number of bubbles on cell membranes after hypoglycaemic and hypoxic stimulation was reduced by ChemR23 overexpression. However, LY294002 or ML385 treatment increased the number of bubbles, indicating that the PI3K and Nrf2 pathways are critical in pyroptosis inhibition by ChemR23 activation (Fig. 8A). Similar results were also found by LDH assay. LDH release in the cell culture supernatant was significantly reduced by ChemR23 overexpression, while LY294002 or ML385 treatment abolished these effects (Fig. 8C). Additionally, the western blot and ELISA results also indicated that LY294002 and ML385 abolished the protective effects of ChemR23 overexpression. As depicted in Fig. 8D–J, ChemR23 overexpression significantly decreased the expression of IL-1β, IL-18, NLRP3, GSDMD-N, ASC and caspase-1 p20 following low glucose and hypoxia stimulation, while administration of LY294002 and ML385 prohibited these effects. Similar changes in the protein levels of NLRP3 were also observed by immunofluorescence staining (Fig. 8B). We further validated the mechanisms of ChemR23 signalling on the PI3K/AKT/Nrf2 pathway in vitro by western blotting. As indicated in Fig. 8K–N, the expression levels of p-PI3K/PI3K, p-AKT/AKT and Nrf2 were all significantly increased in ChemR23 overexpressing cells under hypoglycaemic and hypoxic conditions. On the other hand, incubation with LY294002 significantly inhibited the increase in p-PI3K/PI3K, p-AKT/AKT and Nrf2 (Fig. 8K–N). Moreover, compared to treatment with ChemR23 overexpression alone, ML385 treatment decreased the protein levels of Nrf2 without changing the levels of p-PI3K/PI3K or p-AKT/AKT (Fig. 8K–N). Taken together, these in vitro results confirmed that ChemR23 activation exerts protective effects against pyroptosis through the PI3K/AKT/Nrf2 pathway.

## Discussion

**Fig. 9:**
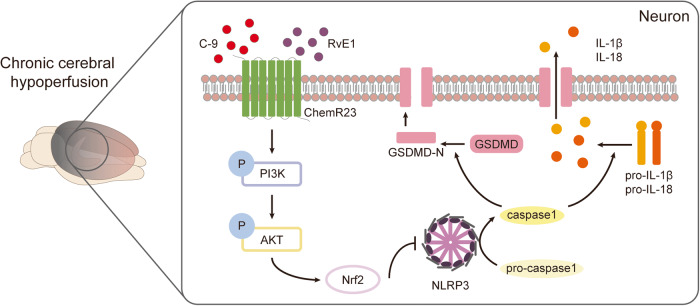
Schematic illustration of ChemR23 activation inhibiting CCH-induced neuronal pyroptosis via the PI3K/AKT/Nrf2 signalling pathway. ChemR23 expression was decreased after CCH. The activation of ChemR23 by RvE1 or C-9 increased the phosphorylation of the PI3K/AKT pathway, which in turn increased Nrf2 levels and ultimately ameliorated NLRP3-related pyroptosis.

In this study, we explored the protective effects of ChemR23 activation and its potential mechanism in CCH models in vivo and in vitro. First, we showed that in response to CCH, hippocampal neurons underwent NLRP3 inflammasome-mediated pyroptosis, leading to neuronal damage, synaptic dysfunction and ultimately cognitive impairment. Second, we found that ChemR23 expression was reduced in CCH rats, and treatment with the ChemR23 agonists RvE1 or C-9 prevented the above CCH-induced impairments. Third, we demonstrated that ChemR23 activation or overexpression also inhibited NLRP3 inflammasome-mediated neuronal pyroptosis under hypoglycaemic and hypoxic condition in vitro, whereas PI3K and Nrf2 inhibitors abolished the protective effects of ChemR23 activation. Collectively, these data are the first to demonstrate that ChemR23 activation improves neuronal pyroptosis in CCH via the PI3K/AKT/Nrf2 pathway, indicating that ChemR23 can serve as an important target for treating CCH-induced VaD (Fig. 9).

Emerging evidence suggests that ChemR23 signalling is an important therapeutic target in various neurological disorders [10, 27, 28]. It has been reported that chemerin administration suppresses neuroinflammation and improves neurological recovery after stroke via ChemR23 [29]. Additionally, C-9 treatment could ameliorate amyloid β1–42 (Aβ1–42)-induced neuroinflammation and memory impairment [18]. Furthermore, RvE1 is also a potent anti-inflammatory and pro-resolving mediator that has been shown to activate ChemR23 [28, 30, 31]. In a mouse model of Down syndrome, RvE1 significantly ameliorated neuroinflammation in the hippocampus and prevented cognitive impairment [32]. In addition, RvE1 promoted the resolution of inflammation in LPS-induced microglia in vitro [33]. In our study, we found that the expression of ChemR23 was significantly decreased along with cognitive impairment at the 8th week after CCH, while treatment with either RvE1 or C-9 obviously prevented this impairment. Moreover, it is widely accepted that synaptic impairment is associated with cognitive dysfunction in CCH [21, 22]. We also observed impaired synaptic function of decreased dendritic spine density and reduced expression of SYN and PSD95 in CCH rats, whereas ChemR23 activation by RvE1 or C-9 rescued synaptic function.

Pyroptosis is a pro-inflammatory form of programmed cell death initiated by the assembly of various inflammasomes, leading to cell lysis and the release of pro-inflammatory cytokines [34]. NLRP3 inflammasome-mediated neuronal pyroptosis and neuroinflammation have been demonstrated to be involved in the development of various neurological diseases, including AD, cerebral ischaemia-reperfusion injury and diabetes-induced brain injury [6, 28, 35]. Accumulating studies have suggested that neuroinflammation can lead to neuronal damage, synaptic injury and cognitive deficits in CCH [3, 9], and it has also been reported that the levels of the NLRP3 inflammasome are significantly increased in CCH [5, 36, 37]. Consistently, we found that the expression levels of pyroptosis-related proteins were elevated in the hippocampus of CCH rats. Moreover, an increase in GSDMD membrane pore formation in hippocampal neurons was also observed by TEM, further confirming the activation of neuronal pyroptosis in CCH rats. In agreement with the results of the in vivo experiments, an increase in pyroptosis-related proteins was also detected in primary hippocampal neurons and SH-SY5Y cells under hypoxic or hypoglycaemic and hypoxic condition in vitro. On the other hand, administration of RvE1 or C-9 inhibited neuronal pyroptosis in CCH in vivo and in vitro. Since RvE1 and C-9 may also bind to other receptors in addition to ChemR23 [38, 39], we used α-NETA, a ChemR23 inhibitor [27], to examine the mechanisms of RvE1 and C-9 treatment in vitro. We found that the protective effects of RvE1 and C-9 on neuronal pyroptosis were significantly abrogated by α-NETA, suggesting that the beneficial effects of RvE1 and C-9 were ChemR23 dependent. These data showed that neurons underwent pyroptosis in CCH and that ChemR23 activation through RvE1 or C-9 treatment rescued neuronal pyroptosis and improved cognitive impairment. The expression of autophagy-related proteins was also assessed in our study. The results indicated that the therapeutic effects of ChemR23 activation also included the improvement of autophagy function in CCH rats. It has been previously reported that improving autophagy function inhibits NLRP3 inflammasome activation in CCH rats [5]. In addition, the NLRP3 inflammasome can also regulate autophagy [40]. Thus, the mechanisms of the interactions between ChemR23 activation, autophagy and pyroptosis need to be investigated in further studies. The molecular pathway by which ChemR23 activation inhibits pyroptosis was further explored. KEGG analysis in the RNA-seq assay showed that most of the differentially expressed genes were enriched in the PI3K/AKT signalling pathway, which plays an important role in multiple cell functions, including cell proliferation and survival [41]. It was previously reported that PI3K/AKT signalling is a downstream pathway of the RvE1/ChemR23 axis [42] and plays a protective role in CCH-induced cognitive impairment [43]. Notably, the activation of this pathway is also closely related to the inflammatory response and pyroptosis. For example, Lu et al. showed that activation of PI3K/AKT signalling improved inflammatory resolution in cerebral ischaemia-reperfusion injury [44]. Another study demonstrated that PI3K/AKT signalling inhibited the NLRP3 inflammasome via Nrf2 activation in a model of hypoxic-ischaemic brain injury [24]. Nrf2 is a key transcription factor that regulates the inflammatory response, maintains cellular redox homoeostasis and plays a neuroprotective role in CCH [45, 46]. Moreover, Nrf2 is also involved in the activation of the NLRP3 inflammasome [47, 48]. Intriguingly, numerous studies have shown that the PI3K/AKT signalling pathway is associated with Nrf2 activation [24–26]. Thus, we tested the hypothesis that ChemR23 signalling activated the PI3K/AKT/Nrf2 pathway and conferred neuroprotective effects in CCH. In our study, SH-SY5Y cells under hypoglycaemic and hypoxic stimulation were used as an in vitro model for CCH [49]. Consistent with the in vivo experiments, we found that treatment with RvE1 or C-9 and ChemR23 overexpression decreased the expression levels of pyroptosis-related proteins, and increased the phosphorylation of PI3K/AKT and the expression of Nrf2. Furthermore, the effects of ChemR23 overexpression were abolished by treatment with PI3K/AKT or Nrf2 inhibitors. These data from in vitro experiments demonstrated that the PI3K/AKT/Nrf2 pathway was essential for ChemR23 activation to inhibit neuronal pyroptosis in CCH.

In conclusion, we proved for the first time that ChemR23 activation with RvE1 or C-9 inhibits neuronal pyroptosis and improves cognitive function in CCH, and the underlying mechanism is through the activation of the PI3K/AKT/Nrf2 pathway. Altogether, our findings suggest that ChmR23 is a promising therapeutic target to treat CCH-induced cognitive impairment.

## Materials and methods

### Animals

Six-week-old male Sprague‒Dawley rats (160–180 g) were purchased from the Experimental Animal Centre of Shanghai Sixth People’s Hospital Affiliated to Shanghai Jiao Tong University School of Medicine (Shanghai, China) and housed in specific pathogen-free conditions with controlled room temperature (20–25 °C), humidity (50–60%), and light (12 h light/12 h dark cycle). The animals had free access to food and water. All experimental procedures in this study were performed in accordance with the principles outlined in the National Institutes of Health (NIH) Guide for the Care and Use of Laboratory Animals and approved by the ethical committee on animal welfare of Shanghai Sixth People’s Hospital Affiliated to Shanghai Jiao Tong University School of Medicine.

### CCH rat model and drug administration

Rats were randomly divided into 4 groups as follows (*n* = 12 in each group): (1) sham-operated + normal saline (sham), (2) bilateral common carotid artery occlusion (BCCAO) + normal saline (CCH), (3) BCCAO + RvE1 (RvE1), (4) BCCAO + C-9 (C-9). BCCAO surgery was performed as described previously [3]. Rats were anaesthetized with sodium pentobarbital (50 mg/kg, i.p.), and then, a ventral midline incision was made. After exposing the bilateral carotid sheaths and carefully separating the common carotid arteries, each common carotid artery was doubly ligated with a 4-0 silk suture and cut off between the ligations. Sham-operated rats underwent the same procedure but without ligation. RvE1 (6 µg/kg, No. 10007848, Cayman Chemical, Michigan, USA) and C-9 (60 µg/kg, No. 7117/1, Tocris Bioscience, Abingdon, UK) were diluted in sterile phosphate-buffered saline (PBS) and injected intraperitoneally every other day for 4 weeks from the 8th week after BCCAO surgery. Additionally, the sham and BCCAO groups received comparable volume injections of PBS as the vehicle. A schematic diagram of the experimental procedure can be found in Fig. 1A. The dosages of RvE1 and C-9 above were selected based on previous studies [27, 32, 50].

### Laser speckle contrast imaging

Following BCCAO surgery, rats were placed in the stereotaxic frame with the head fixed to avoid motion artefacts during imaging. Then, the skull was thinned and polished using a 1.5 mm diameter drill from the coronal line of the fontanelle to the coronal line of the lambdoid suture until the vasculature was obscurely visible. PBS was applied to the thinned skull surface to ensure clear imaging. Rats were then moved to the laser probe for the following assays. Changes in cortical blood flow before and after BCCAO surgery were measured by the laser speckle contrast imager (PeriCam PSI HD system, Perimed, Datavägen 9 A, Sweden). The entire polished brain area was taken as the region of interest and each rat was monitored for 5 min.

### Morris water maze test

At the end of 4 weeks of RvE1 and C-9 treatment, spatial learning and memory were assessed using the Morris water maze (MWM) as described previously [3]. A circular pool (150 cm in diameter, 50 cm in height) filled with water at 23 ± 1 °C to a depth of 45 cm was divided into 4 quadrants, and a platform submerged 2 cm below the water surface was placed in the centre of the 3rd quadrant. Visual cues were set in each quadrant for spatial orientation, and the data were collected using the video tracking system EthoVision® XT 15. During the acquisition test phase, which lasted for 5 days, rats were placed in one of the four quadrants to search for the hidden platform for 60 s. If the rats successfully reached the platform, the time required (escape latency) was recorded; however, if the rats failed to reach the platform, they were then guided to the platform and kept there for 10 s. Four rounds of trials were performed each day, with each round beginning at a different quadrant. The probe trial was performed on the 6th day. Each rat was placed into the first quadrant and swam in the pool for 60 s without the platform. The time each rat spent in the third quadrant and the number of times it crossed the platform were recorded.

### Cell culture and treatment

SH-SY5Y cells (Zqxzbio, Shanghai, China) that had been authenticated and tested for mycoplasma contamination were cultured in high-glucose Dulbecco’s modified Eagle’s medium (DMEM) (4.5 g/L glucose) (Gibco, California, USA) containing 10% foetal bovine serum (Gibco, California, USA) and 100 U/ml penicillin‒streptomycin (Invitrogen, California, USA) at 37 °C and 5% CO2 saturated humidity. On the day before retrovirus transduction, SH-SY5Y cells were plated in a six-well plate and allowed to develop to 40% confluence. On the next day, recombinant lentivirus with the ChemR23-overexpression plasmid (Genomeditech, Shanghai, China) was added to the cells at an MOI of 20. After 24 h of transfection, the cells were cultured with virus-free fresh cell culture medium for another 48 h. Puromycin (6 µg/ml) was subsequently used for the screening of stable transfectants.

To simulate CCH in vitro, a chronic hypoxic and hypoglycaemic culture environment was constructed using low-glucose DMEM (1 g/L glucose) (Gibco, California, USA) and a three-gas incubator (parameters set at 37 °C, 3% O2, 5% CO2, 92% N2). The optimal time of hypoxic and hypoglycaemic stimulation, as well as the optimal treatment concentration of RvE1 and C-9, were determined according to the CCK-8 results. After the above parameters were determined, different combinations of RvE1, C-9, α-NETA (10 µM), LY294002 (20 µM) and ML385 (20 µM) were used in different treatment groups.

Primary hippocampal neurons were prepared from rat embryos at embryonic Day 17 (E17). The foetal rat brain was soaked in dissection medium (DMEM/F12, 10% FBS), and the hippocampus was dissected under a microscope. Then, the hippocampus was minced and digested with 2 mg/ml papain (Macklin, Shanghai, China) and 0.1 mg/ml DNase (Macklin, Shanghai, China) (37° for 20 minutes). After terminating the digestion, 50 µg/mL DNase was added, and the hippocampal tissue was triturated gently to obtain a single-cell suspension, which was subsequently layered on top of the OptiPrep density gradient (Serumwerk Bernburg AG, Bernberg, Germany). Purified neurons were obtained after centrifugation at 800 × *g* for 15 min. The purified neurons were resuspended in dissecting medium and plated on poly-L-lysine-coated coverslips (0.1 mg/ml) (Sigma, Missouri, USA) at 7 × 10^5^ cells/cm^2^. After 1.5 h of incubation, the dissecting medium was replaced with neuronal culture medium containing neurobasal medium, GlutaMAX and B-27 (all from Gibco, California, USA). The cells were maintained at 37 °C in 5% CO2. After 7 days of culture, cells were harvested for analytical experiments. In the following experiments, a three-gas incubator (37 °C, 3% O2, 5% CO2, 92% N2) was used to mimic CCH in vitro.

### Transcriptome sequencing analysis

RNA-seq was conducted by LC-BIO (Hangzhou, China). Total RNA was extracted from the hippocampus of rats to construct a cDNA library and perform RNA-seq. The expression levels of genes were calculated by quantifying the cDNA fragments per kilobase of transcript per million fragments mapped. Differentially expressed genes (DEGs) between different groups were subjected to Gene Ontology (GO) and Kyoto Encyclopaedia of Genes and Genomes (KEGG) pathway enrichment functional analysis.

### Western blot analysis

Hippocampal tissues and SH-SY5Y cells were homogenized in cold RIPA buffer (Beyotime, Shanghai, China) containing protease and phosphatase inhibitors, followed by centrifugation at 12,000 rpm for 30 min at 4 °C. The protein concentrations were assayed using a BCA kit (Epizyme, Shanghai, Shanghai). Different samples with the same amount of protein were separated by sodium dodecyl sulphate‒polyacrylamide gel electrophoresis (SDS‒PAGE) on 7.5% or 12% gels (Epizyme, Shanghai, Shanghai) and transferred to polyvinylidene difluoride membranes (PVDF). The membranes were then blocked with 5% BSA for 1 h at room temperature and incubated overnight at 4°C with primary antibodies as follows: synaptophysin (SYN) (1:1000, A6344, ABclonal, Wuhan, China), postsynaptic density protein-95 (PSD95) (1:1000, A7889, ABclonal, Wuhan, China), NLRP3 (1:500, GB114320, Servicebio, Wuhan, USA), GSDMD (1:1000, GB114198, Servicebio, Wuhan, China), ASC (1:1000, A16672, ABclonal, Wuhan, China), caspase1-p20 (1:1000, A16792, ABclonal, Wuhan, China), Bcl-2 (1:1000, A0208, ABclonal, Wuhan, China), Bax (1:1000, A0207, ABclonal, Wuhan, China), LC3B (1:1000, A7198, ABclonal, Wuhan, China), SQSTM1/p62 (1:1000, A7758, ABclonal, Wuhan, China), Nrf2 (1:1000, GB113808, Servicebio, Wuhan, China), CMKLR1 (1:1000, No. 10325, Cayman, Michigan, USA), p-PI3K (1:1000, AP0854, ABclonal, Wuhan, China), PI3K (1:1000, A0982, ABclonal, Wuhan, China), p-AKT (1:1000, AP0140, ABclonal, Wuhan, China), AKT (1:1000, A17909, ABclonal, Wuhan, China) and β-actin (1:1000, 3700, CST, Massachusetts, USA). The membranes were washed in TBST and incubated with relevant secondary antibodies at room temperature for 1 h. Then, immunoblots were detected using the ECL kit (Yeasen, Shanghai, China) and analysed by ImageJ software.

### Enzyme-linked immunosorbent assay (ELISA)

The levels of IL-1β (Multisciences, Hangzhou, China) and IL-18 (Thermo Fisher Scientific, Massachusetts, USA) in hippocampal tissues and culture media were determined using ELISA kits according to the manufacturer’s instructions.

### H&E and Nissl staining

The rat brain fixed in 4% formalin was cleaned, dehydrated, paraffin-embedded, and cut into 6-μm thick coronal slices for haematoxylin and eosin (H&E) and Nissl staining. The brain sections were stained with haematoxylin and eosin or 0.5% tolyl violet solution, followed by dehydration with graded ethanol, transparentization with xylene, and finally coverage with neutral resin [51]. The morphological damage and neuronal injuries were observed by an optical microscope (IX53, Olympus, Tokyo, Japan).

### Golgi staining

Golgi staining was performed using a Rapid GolgiStain Kit (FDneurotech, USA) according to the manufacturer’s instructions. After perfusion with PBS for 5 min, the brain tissue was immersed in impregnation solution, which was replaced every day, and stored at room temperature in the dark for 4 weeks. Then, the brain tissue was transferred to Solution C and stored at 4 °C for another 72 h. A vibrating microtome (VT 1000 s, Leica, Germany) was used to prepare the brain sections (100 μm in thickness). Next, the sections were dehydrated in ethanol and cleared in xylene. The spine morphology of neurons was observed and analysed using the microscope (BX53, Olympus, Tokyo, Japan).

### Immunofluorescence staining

For in vivo fluorescence staining, after deparaffinization and antigen retrieval (0.05% citraconic acid), the paraffin-embedded sections were treated with endogenous peroxidase (3% H_2_O_2_ in PBS) for 10 min, followed by incubation with 5% donkey serum containing 0.1% Triton X-100 in PBS for 1 h. Next, the sections were incubated overnight at 4 °C with the following primary antibodies: CMKLR1 (1:200, No. 10325, Cayman, Michigan, USA), NLRP3 (1:500, BA3677, Boster, Wuhan, China), GSDMD (1:500, A22523, ABclonal, Wuhan, China), PSD95 (1:100, 17785-1-AP, Proteintech, Wuhan, China), SYN (1:400, 17785-1-AP, Proteintech, Wuhan, China), GFAP (1:200, 60190-1-Ig, Proteintech, Wuhan, China), Iba-1 (1:500, 66827-1-Ig, Proteintech, Wuhan, China) and NeuN (1:100, 66836-1-Ig, Proteintech, Wuhan, China), followed by incubation with the corresponding fluorescent secondary antibody the next day. Nuclei were stained with 4′,6-diamidino-2-phenylindole (DAPI) for 5 min at room temperature. For in vitro fluorescence staining, cells were fixed in 4% paraformaldehyde for 15 min, followed by permeabilization and blocking with 5% donkey serum containing 0.1% Triton X-100 in PBS for 1 h at room temperature. Next, the cells were incubated with the NLRP3 (1:200, DF15549, Affinity, Ohio, USA) primary antibody overnight at 4 °C and subsequently incubated with the corresponding secondary antibody for 1 h at room temperature. Next, the cells were incubated overnight at 4 °C with primary antibodies as follows: NLRP3 (1:200, DF15549, Affinity, Ohio, USA), GSDMD (1:100, GB114198, Servicebio, Wuhan, China) and MAP2 (1:200, 67015-1-Ig, Proteintech, Wuhan, China). The cells were then incubated with secondary antibodies for 1 h at room temperature. Images were acquired using a fluorescence microscope (IX53, Olympus, Tokyo, Japan) or a confocal microscope (Nikon, Tokyo, Japan).

### Transmission electron microscopy (TEM)

According to our previously published method [3], the CA1 subregion of the hippocampus was dissected and cut into 1 mm sections as soon as possible after removing the brain. Then, after fixation in electron microscopy fixative (Servicebio, China) overnight at 4 °C, the sections were washed with 0.1 M PBS and postfixed with OsO4 for 2 h at room temperature. Next, the blocks were dehydrated by graded ethanol, embedded in resin, and eventually cut into ultrathin slices (60-80 nm), which were stained with uranyl acetate and lead citrate. The slices were then analysed using a transmission electron microscope (H-7800, Hitachi, Japan).

### Calcein-AM and propidium iodide (PI) assays

To visualize the effects of different treatments on cell viability, the cells were stained with a live-dead staining kit (Yeasen Biotech, Shanghai, China). According to the manufacturer’s protocol, the cultured cells were incubated with Calcein-AM and PI solution for 15 min at 37 °C. The results were observed under a fluorescence microscope (IX53, Olympus, Tokyo, Japan). Viable cells were stained green with Calcein AM and the nuclei of dead cells were stained red with PI.

### Cell viability assays

Cell viability was assessed by Cell Counting Kit-8 (Beyotime, China) according to the manufacturer’s instructions. SH-SY5Y cells were seeded onto 96-well plates and cultured overnight, followed by different treatments. After the treatments, 10 μl of CCK-8 solution was added to each well and incubated for 1 h at 37 °C. The absorbance of each well was detected at 450 nm under a microplate reader (Thermo Fisher Scientific, USA), and the cell viability was calculated.

### LDH release assay

Lactate dehydrogenase (LDH) released into the cell culture supernatants after different treatments was detected using an LDH Cytotoxicity Assay Kit (Beyotime, Shanghai, China) according to the manufacturer’s protocol. The absorbance at 490 nm was detected using a microplate reader (Thermo Fisher Scientific, USA).

### Annexin V-FITC and PI dual staining assay

Cell death was detected using an annexin V-fluorescein isothiocyanate (FITC) and propidium iodide (PI) Apoptosis Detection Kit (Dojindo, China) according to the manufacturer’s instructions. After different treatments, adherent and floating cells were collected, washed twice with PBS, and incubated in annexin V and PI for 15 min at room temperature in the dark. At least 1.0 × 10^4^ cells were collected and detected with a flow cytometer (FACSVerse, Bioscience, USA), and the data were analysed by FlowJo software.

### Scanning electron microscopy (SEM)

According to the protocol of a previous study [52], cells after different treatments were digested, centrifuged, and resuspended with an electron microscope fixative (Servicebio, China). After fixing at room temperature for 2 h, the cells were dehydrated through a graded series of ethanol and dried by the tertiary butanol method. Next, the cells were sputter coated with gold and examined under the Hitachi S-4800 scanning electron microscope (SEM) (Hitachi, Ltd., Japan).

### Statistical analysis

The statistical analyses were performed with GraphPad Prism 8 software. The differences among groups were analysed by one-way or two-way analysis of variance (ANOVA) with Tukey’s post hoc test or Student’s t test. The data are expressed as the mean ± standard error of the mean (SEM). *P* value < 0.05 was considered to be statistically significant.

### Supplementary information

Supplementary Information

Figure S1

Figure S2

Figure S3

Figure S4

Original Data File

Reproducibility checklist

## Data Availability

The datasets generated during and/or analysed during the current study are available in the SRA repository. https://www.ncbi.nlm.nih.gov/sra/PRJNA1004467
