# Supplementary material for: ChemR23 activation attenuates cognitive impairment in chronic cerebral hypoperfusion by inhibiting NLRP3 inflammasome-induced neuronal pyroptosis
Source: Cell Death Dis. 2023 Nov 6;14(11):721. doi: 10.1038/s41419-023-06237-6 (PMC10628255; doi:10.1038/s41419-023-06237-6)
Supplement: Supplementary file 1 — Supplementary Information [file 41419_2023_6237_MOESM1_ESM.docx]

**Supplementary Information**

**Figure S1. Expression of ChemR23 in neurons, microglia and astrocytes.** (A) Representative merged immunofluorescence images of NeuN/ChemR23/DAPI in the CA1 region of rats 12w after the surgery. Scale bar, 20 μm. n = 3 for each group. (B) Representative merged immunofluorescence images of Iba-1/ChemR23/DAPI in the CA1 region of rats 12w after the surgery. Scale bar, 20 μm. n = 3 for each group. (C) Representative merged immunofluorescence images of GFAP/ChemR23/DAPI in the CA1 region of rats 12w after the surgery. Scale bar, 20 μm. n = 3 for each group.

**Figure S2. Expression of ChemR23 in hippocampal CA1, CA2 and DG regions.** (A) Representative merged immunofluorescence images of NeuN/ChemR23/DAPI in the CA2, CA3 and DG region of rats. Scale bar, 20 μm. n = 3 for each group.

**Figure S3. Activation of ChemR23 with RvE1 or C-9 ameliorates the reduction of PSD-95 and SYN expression after CCH.** (A) Representative immunofluorescence staining images of PSD-95/DAPI in the CA1 region of rats from Sham, CCH, RvE1 and C-9 groups. n = 3 for each group. (B) Representative immunofluorescence staining images of SYN/DAPI in the CA1 region of rats from sham, CCH, RvE1 and C-9 groups. n = 3 for each group. (C) Mean fluorescence intensity of PSD-95 in each group. n = 3 for each group. (D) Mean fluorescence intensity of SYN in each group. n = 3 for each group. Data are presented as the mean ± SEM. ***P < 0.001 vs. Con; ##P < 0.01, ###P < 0.001 vs. CCH

**Figure S4. Effects of ChemR23 activation on markers of apoptosis and autophagy in CCH rats.** (A-C) Representative immunoblotting and semi-quantification of apoptosis-related protein, Bcl-2 and Bax, in each group. n = 4 for each group. (D-F) Representative immunoblotting and semi-quantification of autophagy-related protein, p62 and LC3B-II, in each group. n = 4 for each group. Data are presented as the mean ± SEM. ***P < 0.001 vs. Con; #P < 0.05, ##P < 0.01 vs. CCH.
