# Supplementary figures and images for: ChemR23 activation attenuates cognitive impairment in chronic cerebral hypoperfusion by inhibiting NLRP3 inflammasome-induced neuronal pyroptosis
Source: Cell Death Dis. 2023 Nov 6;14(11):721. doi: 10.1038/s41419-023-06237-6 (PMC10628255; doi:10.1038/s41419-023-06237-6)

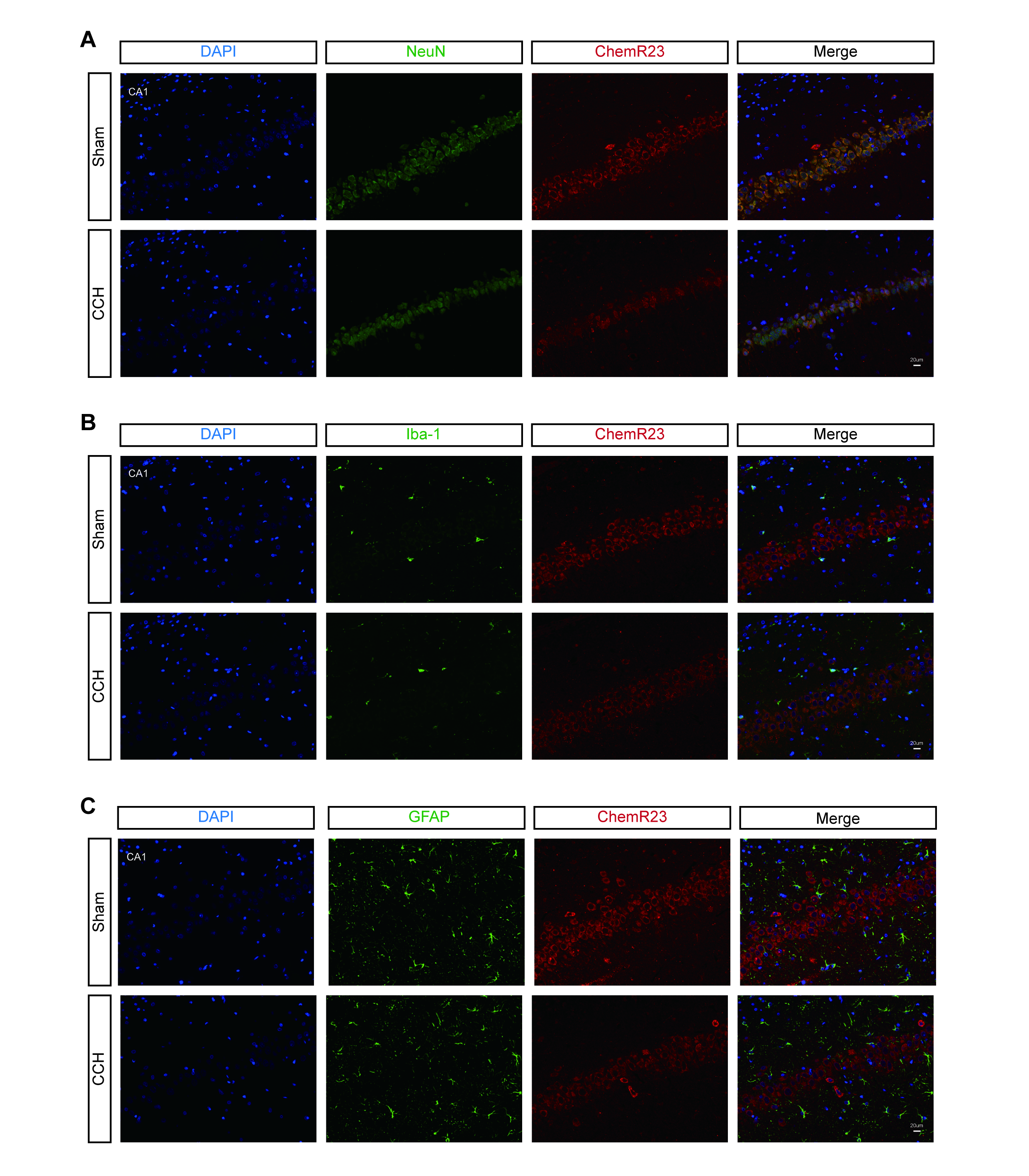

Supplement: Supplementary file 2 — Figure S1 [file 41419_2023_6237_MOESM2_ESM.tif]

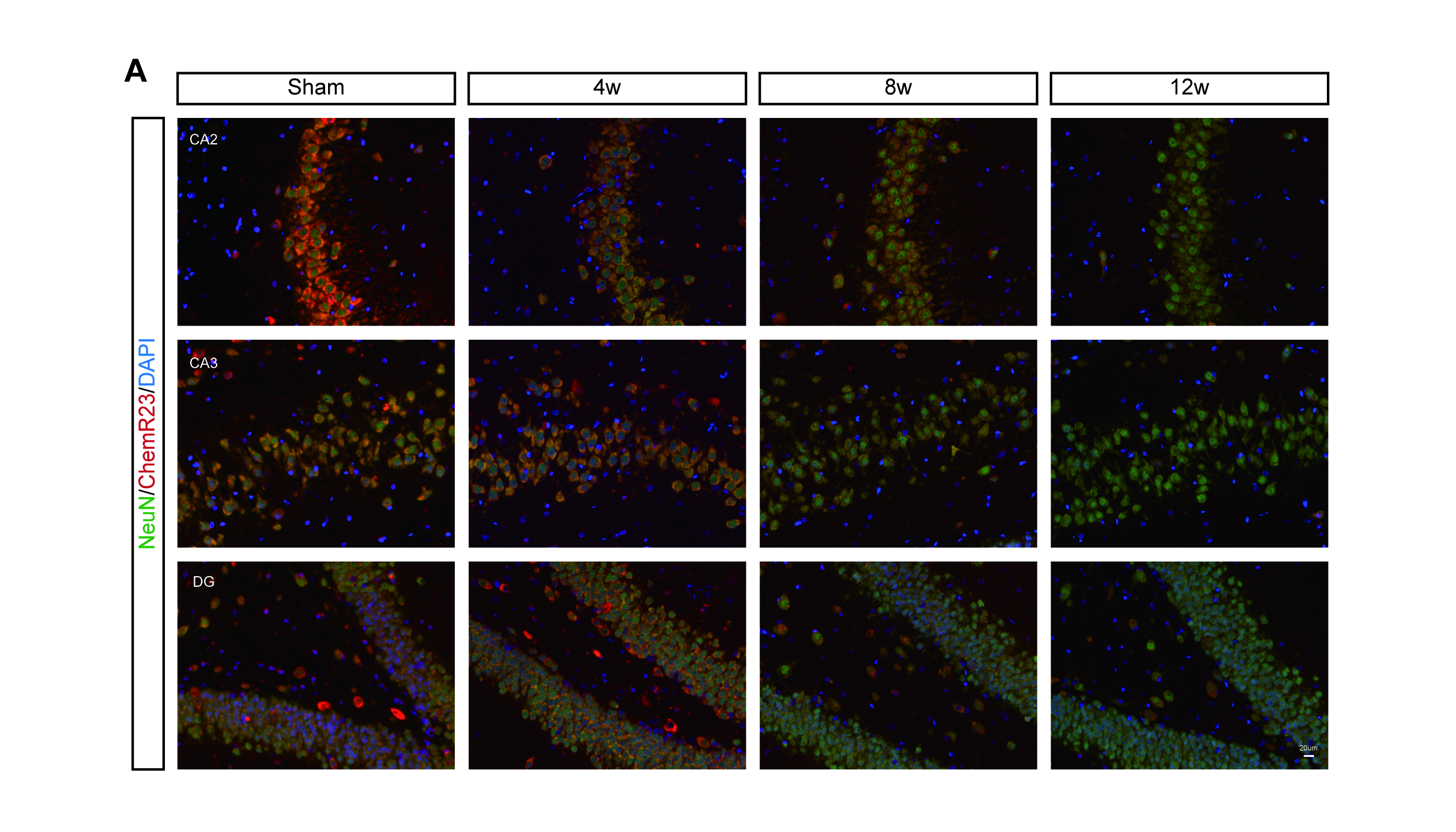

Supplement: Supplementary file 3 — Figure S2 [file 41419_2023_6237_MOESM3_ESM.tif]

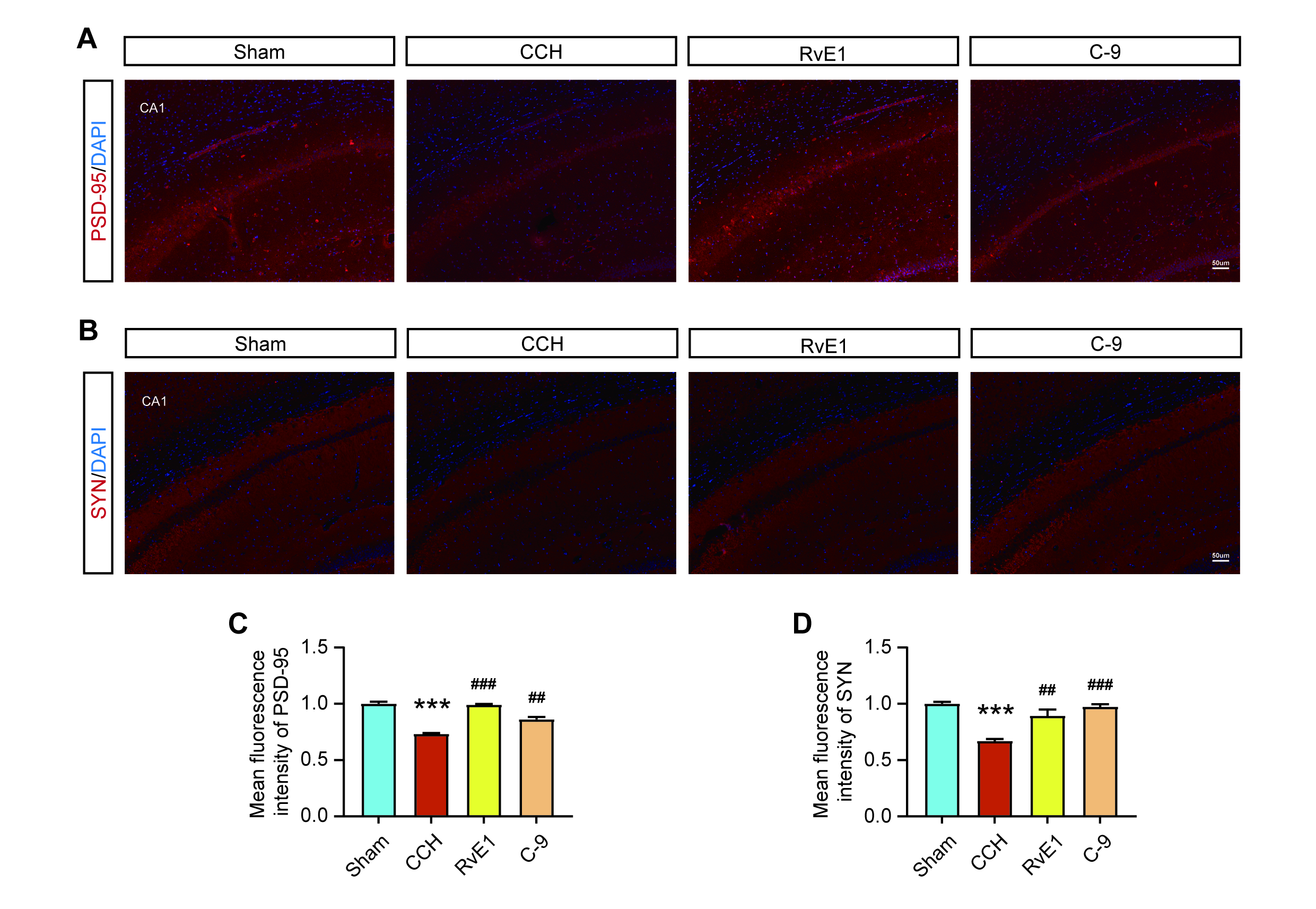

Supplement: Supplementary file 4 — Figure S3 [file 41419_2023_6237_MOESM4_ESM.tif]

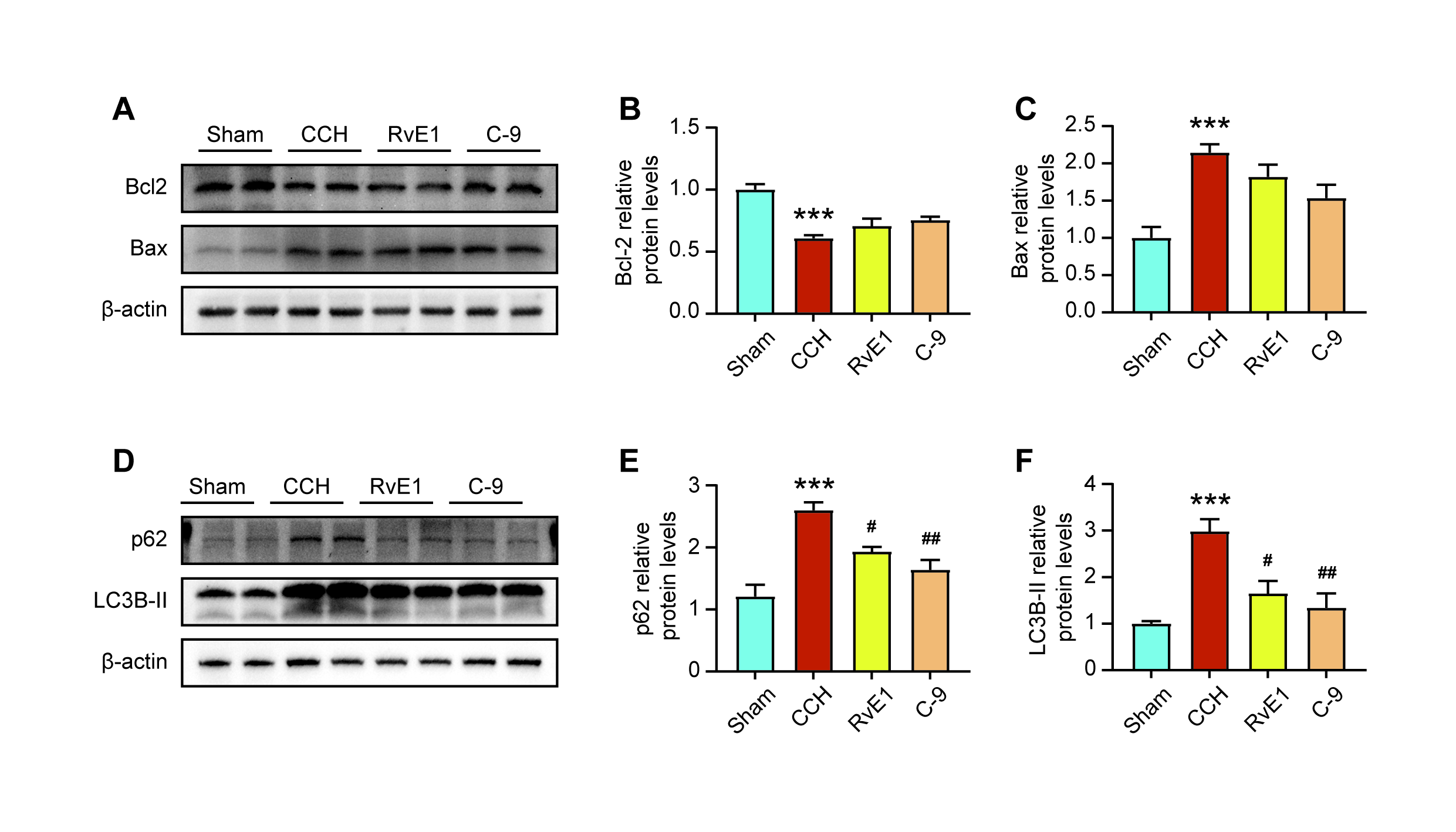

Supplement: Supplementary file 5 — Figure S4 [file 41419_2023_6237_MOESM5_ESM.tif]
