# Supplementary material for: ChemR23 activation attenuates cognitive impairment in chronic cerebral hypoperfusion by inhibiting NLRP3 inflammasome-induced neuronal pyroptosis
Source: Cell Death Dis. 2023 Nov 6;14(11):721. doi: 10.1038/s41419-023-06237-6 (PMC10628255; doi:10.1038/s41419-023-06237-6)
Supplement: Supplementary file 6 — Original Data File [file 41419_2023_6237_MOESM6_ESM.docx]

**Raw image files of Western blots, Some images with unclear edges, we provide the images under white light**

**Figure1**

ChemR23-4w ChemR23-4w-ACTIN


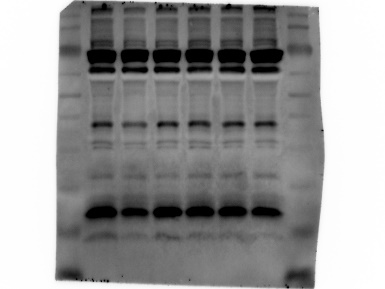



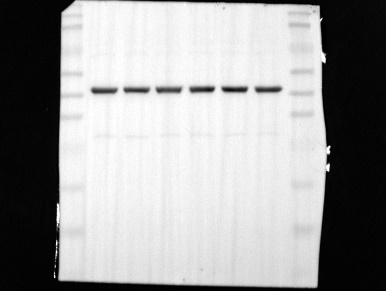


ChemR23-8w ChemR23-8w-ACTIN


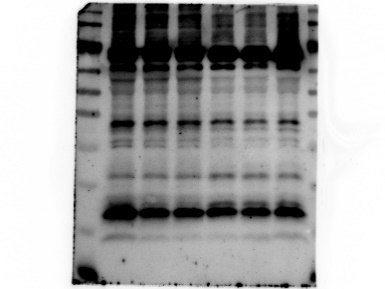



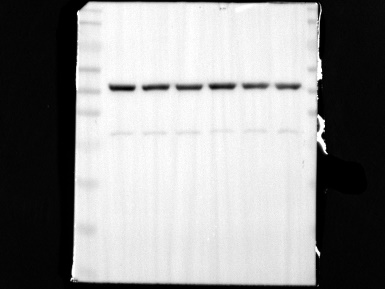


ChemR23-12w ChemR23-12w-ACTIN


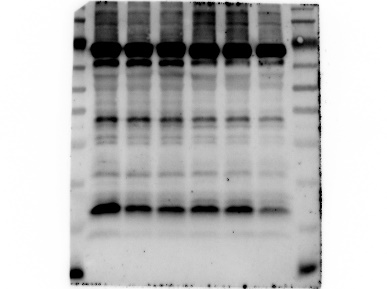



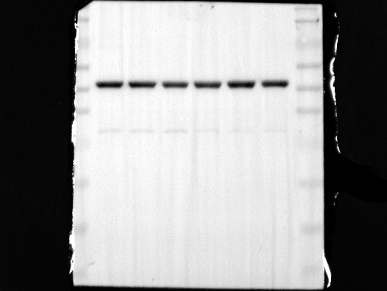


**Figure2**

PSD95 PSD95-ACTIN







SYN SYN-ACTIN




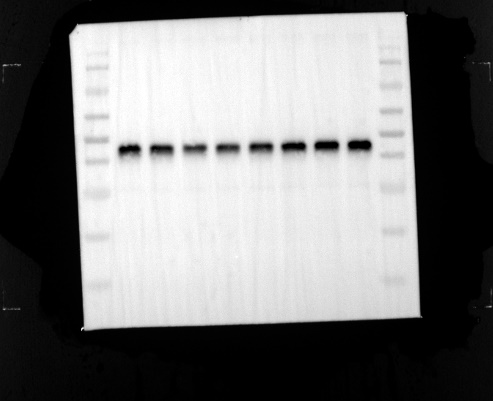



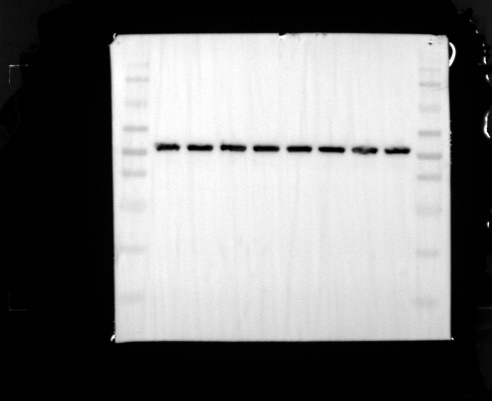


**Figure3**

NLRP3 NLRP3-ACTIN


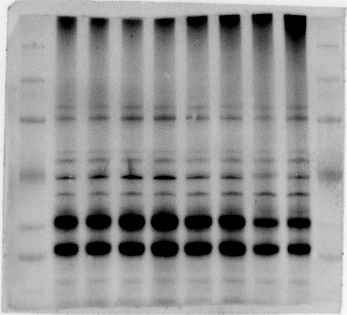



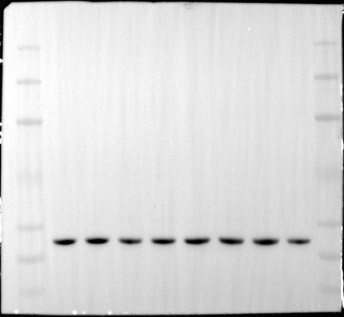


GSDMD-N GSDMD-N-ACTIN


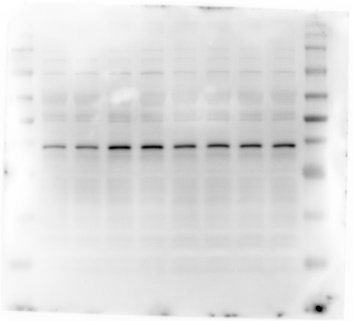




Caspase1-p20 Caspase1-p20-ACTIN


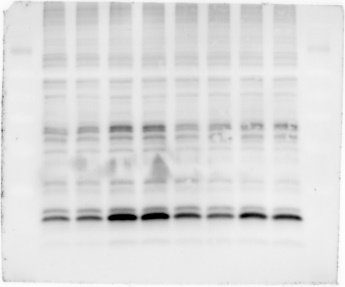



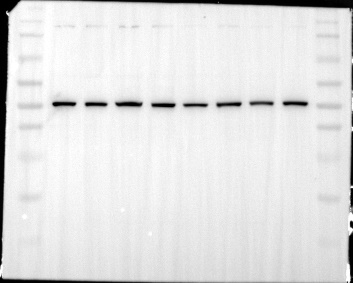


ASC ASC-ACTIN


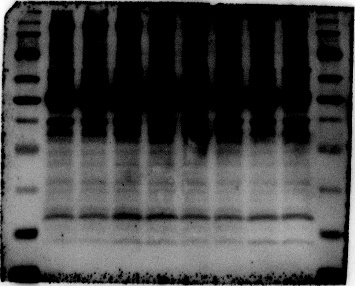

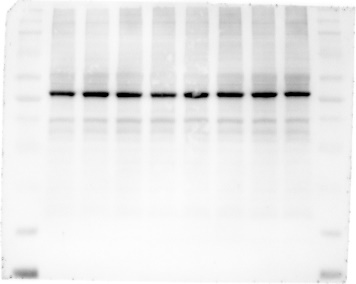


**Figure4**

PI3K p-PI3K ACTIN




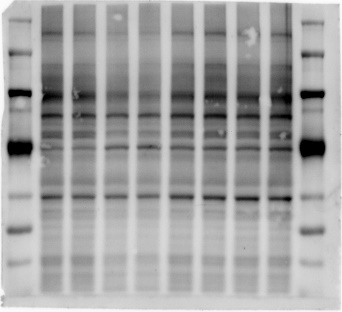

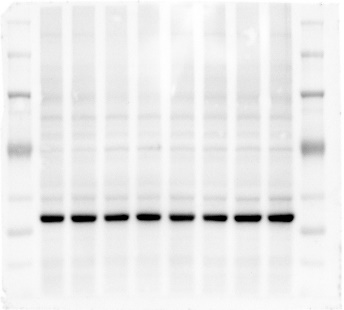


AKT AKT-ACTIN




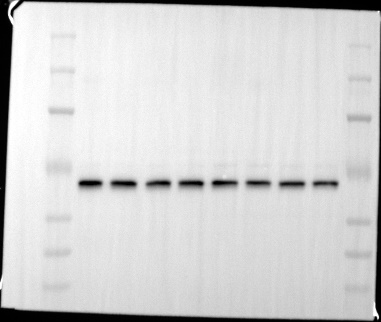



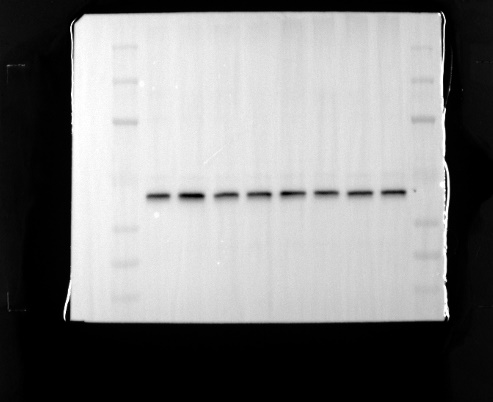


p-AKT p-AKT




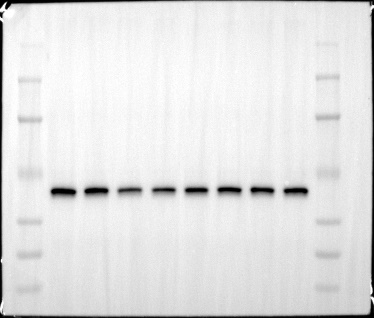



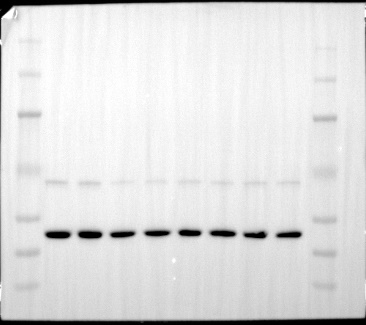


Nrf2 Nrf2-ACTIN


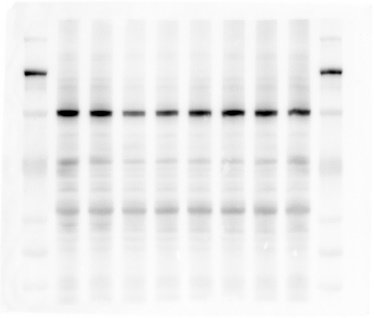

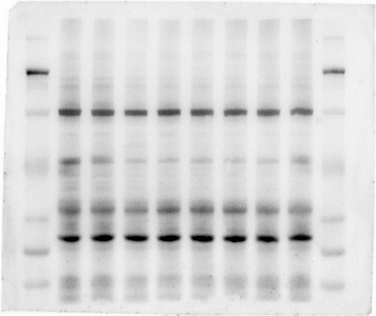


**Figure5**

NLRP3 NLRP3-ACTIN


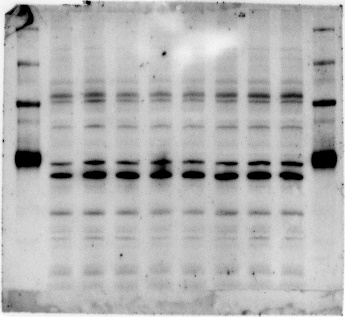

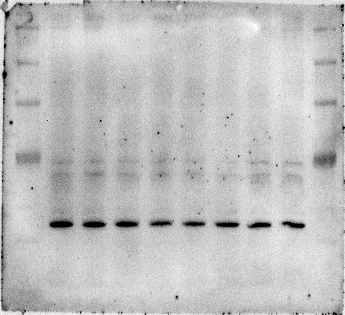


GSDMD-N GSDMD-N-ACTIN


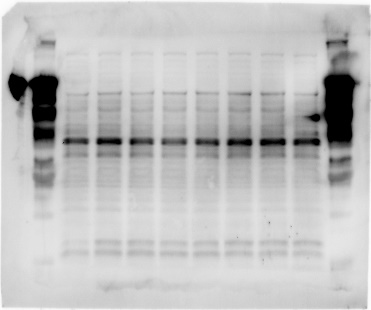

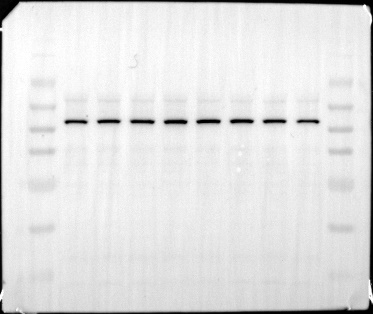


Caspase1-p20 Caspase1-p20-ACTIN


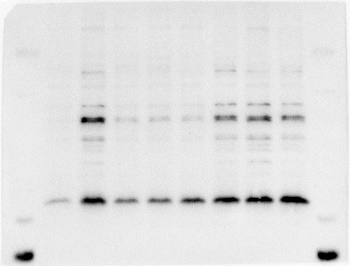



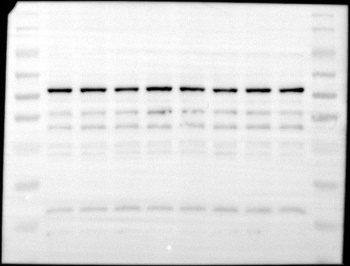


ASC ASC-ACTIN


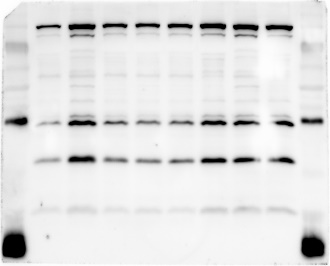



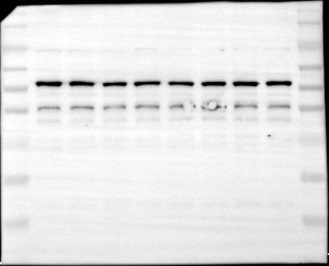


**Figure6**

NLRP3 NLRP3-ACTIN


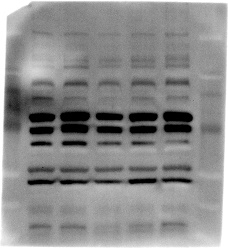

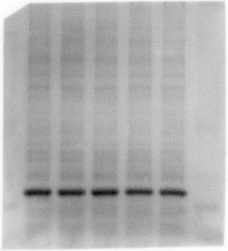


GSDMD-N GSDMD-N-ACTIN


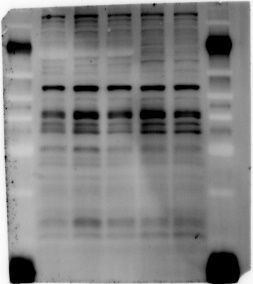



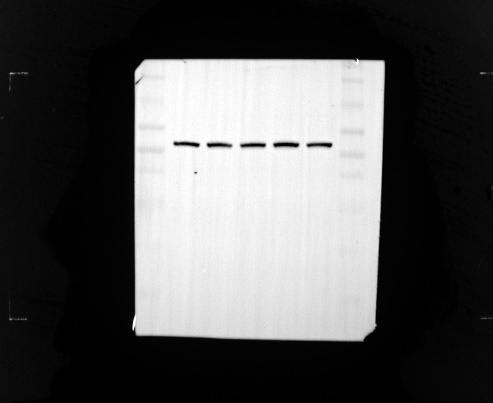


Caspase1-p20 Caspase1-p20-ACTIN


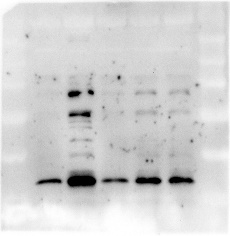

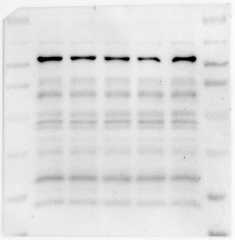


ASC ASC-ACTIN







PI3K p-PI3K PI3K-ACTIN




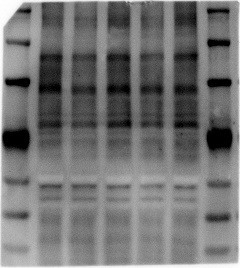




AKT p-AKT AKT-ACTIN


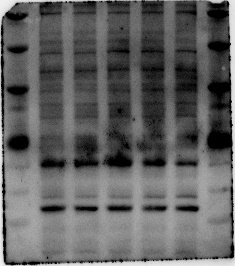






Nrf2 Nrf2-ACTIN


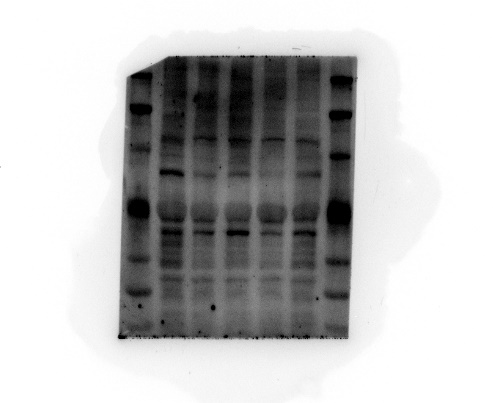




Supplementary Fig. 3

Bcl2 Bcl2-ACTIN


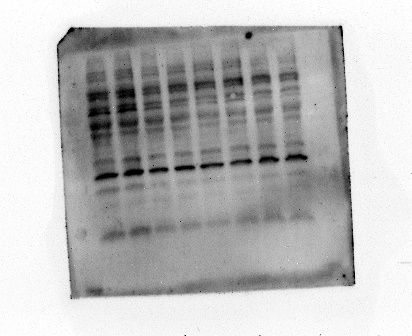




Bax Bax-ACTIN


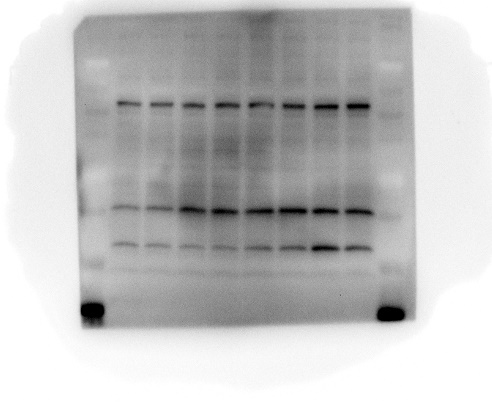




p62 p62-ACTIN


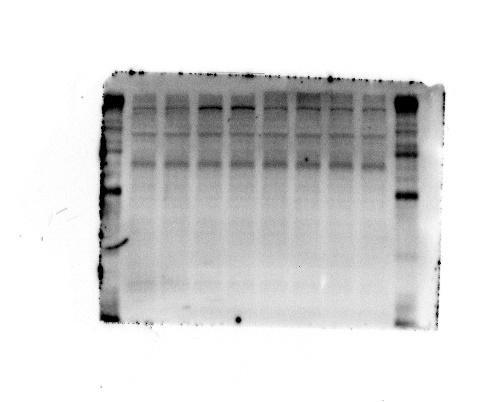






LC3B IC3B-ACTIN
